# Association Between Esports Participation and Health: A Systematic Review and Meta-analysis

**DOI:** 10.1186/s40798-026-01083-0

**Published:** 2026-07-29

**Authors:** Di Tang, Jinde Liu, Pengpeng Gou, Xin Zhang, Chang Liu, Ruisi Ma, Jie Feng, Sara Xiao, Thomas Yuen Tung Lam

**Affiliations:** 1 The Department of Sports Science and Physical Education, Faculty of Education, The Chinese University of Hong Kong, Hong Kong, China; 2 The Nethersole School of Nursing, Faculty of Medicine, The Chinese University of Hong Kong, Hong Kong, China; 3 Faculty of Physical Education, Fudan University, Shanghai, China; 4 Department of Sports and Health Sciences, Faculty of Arts and Social Sciences, Hong Kong Baptist University, Hong Kong, China; 5 Faculty of Education, Central China Normal University, Wuhan, China; 6 Vanke School of Public Health, Tsinghua University, Beijing, China; 7 School of Physical Education, Jinan University, Guangzhou, China; 8 Faculty of Sports Science, Ningbo University, Ningbo, China

**Keywords:** Esports, Health, Video game, Meta-analysis

## Abstract

**Background:**

With the growth of the esports industry, concerns about participants’ health risks have drawn public and academic attention. However, existing research remains inconsistent and often fails to distinguish esports from general video gaming. This study evaluates health differences between esports participants and non-participants and explores correlations between participation and health outcomes.

**Methods:**

A systematic search of seven databases (PubMed, Web of Science, Scopus, SPORTDiscus, MEDLINE, Esports Research Network, and ProQuest) was conducted up to May 13, 2026. Data were synthesized using mean differences (MD) or standardized mean differences (SMD) for group comparisons, and Fisher’s Z-transformed correlation coefficients (r) for relationships between esports participation and health outcomes. Subgroup analyses based on professional and non-professional status were also conducted.

**Results:**

Of 5090 identified studies, 53 (n = 86,652) were included, with 36 analyzed in meta-analyses. Initial analyses showed no significant differences between esports participants and non-participants across physical activity, body mass index (BMI) sleep duration, anxiety, depression, stress, and grip strength. However, following sensitivity analysis, esports participants exhibited significantly lower depression levels (SMD –0.13, 95% CI –0.17 to –0.10). Conversely, non-professional esports players demonstrated a significantly higher body fat percentage than non-players (MD 4.57, 95% CI 1.73 to 7.41). Additionally, esports participation was significantly correlated with sedentary behavior (r = 0.17, 95% CI 0.12 to 0.23), BMI (r = 0.09, 95% CI 0.04 to 0.13), and gaming disorder (r = 0.10, 95% CI 0.05 to 0.15).

**Conclusion:**

Although current evidence suggests that esports participants do not generally display worse overall health than non-participants, the association between esports participation and health is multifaceted and requires a nuanced perspective. Furthermore, given the existing heterogeneity and the cross-sectional design of the included studies, alongside a moderate risk of bias, these findings warrant careful interpretation. Future research must prioritize standardized definitions, consistent reporting, objective measures, and longitudinal designs to further validate these associations and investigate causal relationships. Concurrently, the industry should develop stratified, evidence-based guidelines to safeguard the long-term well-being of different types of players.

*Registration* CRD42024622812 (PROSPERO).

**Supplementary Information:**

The online version contains supplementary material available at 10.1186/s40798-026-01083-0.

## Key Points

- Based on current evidence, esports participants generally do not exhibit a worse overall health status compared to non-participants, challenging prevailing negative perceptions.
- Esports players may exhibit a trend toward lower depression levels compared to non-players, but these potential mental health findings require further validation.
- Given the identified positive correlations between esports participation and BMI, sedentary behavior, and gaming disorder, future longitudinal studies are essential to determine causal relationships.
- It is imperative to develop specialized prevention and intervention measures for esports-specific health risks, alongside stratified health guidelines tailored to different player types.

**Supplementary Information:**

The online version contains supplementary material available at 10.1186/s40798-026-01083-0.

## Introduction

### The Rise of Esports and Associated Health Concerns

In recent years, the esports industry has experienced unprecedented growth [1, 2]. This expansion manifests not only in participation numbers and economic scale but also in its potential influence on youth lifestyle patterns and the emergence of a professional sector [3]. The inclusion of esports as an official competitive event in the 2023 Asian Games marks a significant milestone in its formal recognition within traditional sports frameworks [4]. This formalization is expected to drive further expansion across all participation levels and attract a highly diverse demographic.

However, the exponential growth of esports participation has generated significant health-related concerns within medical and scientific communities [5–7]. Due to the inherent characteristics involving prolonged electronic device use and sedentary behavior, traditional research paradigms have identified associations with various health issues [8–10]. Although participation volume may vary significantly among casual and semi-professional players [11–13], recent studies report that professional esports players can accumulate an average of 9 h of daily sitting and training [6, 14]. Additionally, this sedentary behavior has been observed to increase even further during competition periods [6]. These concerns are substantiated by established correlations between sedentary behavior and adverse health outcomes, including cardiovascular disease, metabolic disorders, and obesity [15, 16]. Furthermore, observational studies have documented specific health challenges among esports participants, encompassing musculoskeletal issues, visual strain, psychological stress, and sleep disturbances [17–19]. Anthropometric data indicate higher body fat percentages and lower lean mass among participants [20].

### Contradictory Findings and Methodological Limitations

Conversely, emerging research challenges these conventional concerns, demonstrating that esports players often maintain health behaviors comparable to age-matched peers [9, 21, 22]. Comparative studies reveal no significant differences in body composition, musculoskeletal pain, or sleep problems [21, 23]. Furthermore, some professional players even demonstrate elevated physical activity levels compared to population norms [24–26]. Notably, a recent study indicates that esports players might over-report physical activity in self-assessments compared to objective measures like accelerometers, casting doubt on the prevailing narrative that these players maintain high levels of physical activity [11]. While this observation offers a novel perspective on the current contradictory results, it remains to be determined whether these measurement discrepancies stem from inherent biases between subjective and objective tools or if they represent an over-reporting phenomenon unique to the esports population in the absence of direct comparative evidence [27, 28]. Overall, these divergent findings suggest a complex relationship between esports and health, likely influenced by participation level, training regimens, and individual lifestyles, which is further complicated by inconsistent population definitions and study heterogeneity that limit comparability.

Additionally, inherent methodological limitations within the primary studies on esports health may also account for these contradictory conclusions [3, 21, 22, 29]. A primary challenge lies in the inconsistencies between the conceptualization and operationalization of esports [29]. Although Hamari and Sjöblom’s definition of esports as “a form of sports where the primary aspects of the sport are facilitated by electronic systems; the input of players and teams as well as the output of the esports system are mediated by human–computer interfaces” has gained widespread acceptance [2], significant heterogeneity remains in how researchers operationalize it in practice.

First, there is a frequent conflation of esports with general video gaming. Although the two have been academically delineated as fundamentally distinct activities [29–31], some existing studies erroneously include general video games lacking fair competitive mechanisms (such as those centered on role-playing or virtual-world exploration) within the scope of esports discussions [3, 32, 33]. Furthermore, many studies fail to explicitly report the genres of games played by participants, lacking a clear operational definition of “esports participation” [34–36]. This conflation likely confounds the distinct health outcomes associated with esports versus general gaming.

Second, there remains a lack of consensus and conceptual ambiguity regarding the exact scope of “esports”. While a broader perspective views esports as a spectrum ranging from recreational to professional levels, there is an ongoing tendency to primarily define it as high-level professional competition [37–39]. Due to this definitional divergence, some studies naturally restrict their samples to professional esports players. However, this conceptual ambiguity introduces a methodological challenge: health outcomes derived from professional cohorts are often generalized to the broader behavior of “esports participation”. In reality, professional athletes face unique, profession-specific health risks stemming from their rigorous training regimens, intense competitive pressures, and career-related stress [40]. Consequently, if the lack of a unified definition fails to clearly distinguish these specific occupational impacts from the health effects of general esports engagement, the specificity and generalizability of the research findings are significantly compromised.

Finally, at the research design level, comparative studies investigating the health status of esports participants against their peers are often limited by insufficient sample sizes, which further undermines the reliability and robustness of existing findings [3, 22].

### Study Rationale and Objectives

**Fig. 1:**
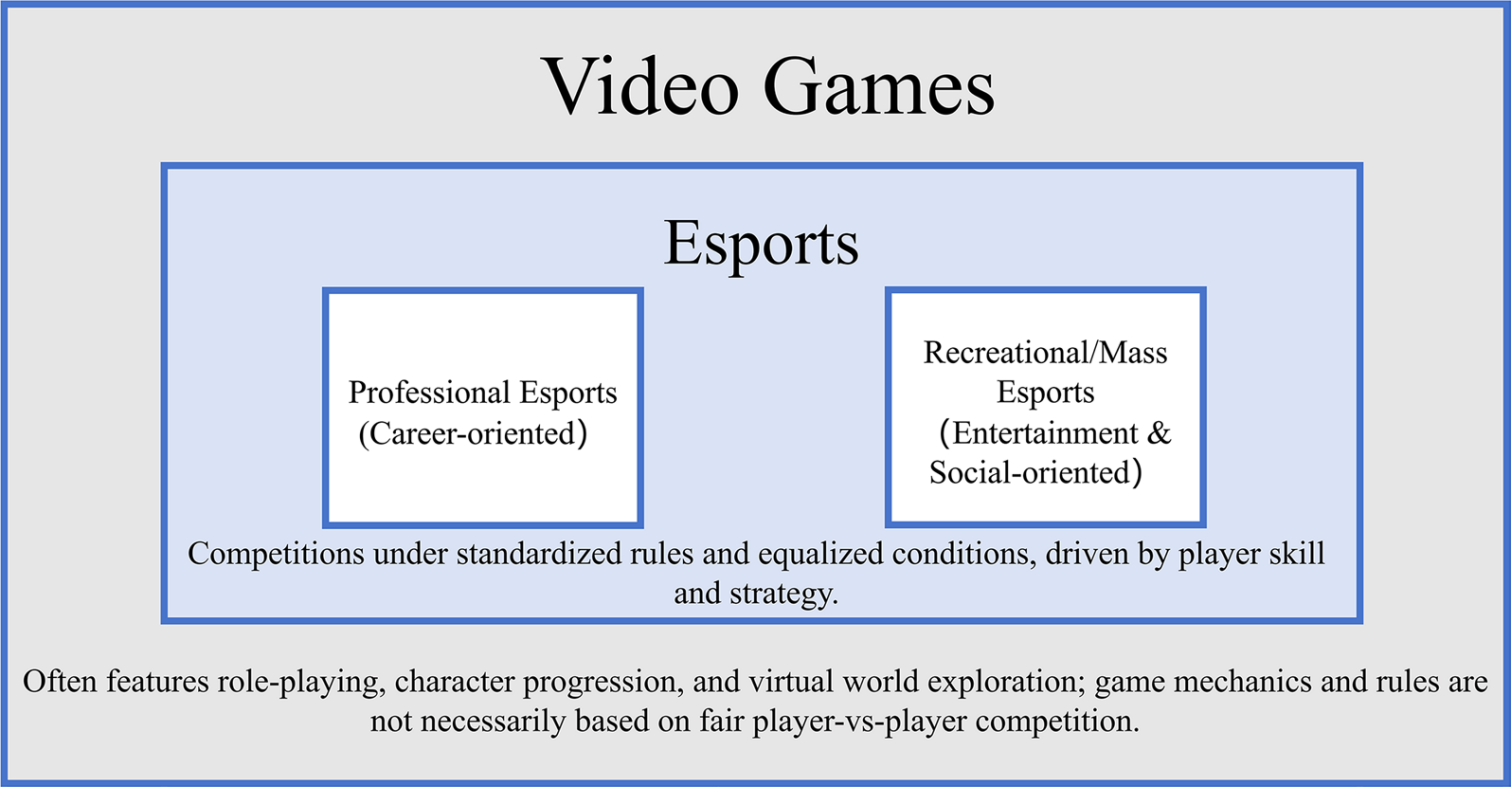
Conceptual boundaries and classification of esports within the context of video gaming. Competition denotes the inherent competitive nature within the game itself, rather than only being strictly defined by externally organized tournaments

Given these methodological limitations and the resulting contradictory findings, a comprehensive and rigorous synthesis of the existing literature is needed to carefully evaluate these associations. Therefore, a systematic review coupled with a meta-analysis of existing empirical studies is highly warranted. While several reviews have explored the relationship between esports participation and health, the majority are scoping reviews or narrative syntheses, lacking an updated and quantitative synthesis with adequate statistical power to measure these associations [1, 22, 41]. In addition, compared to previous reviews, this systematic review will more strictly differentiate between general video gaming and esports participation based on operational definitions, participant criteria, and reported game types in the original articles. To operationalize this differentiation, we adopted a conceptual framework (Fig. 1) based on the theoretical boundaries proposed by a previous definition review [29, 42].

Therefore, the primary objective of this systematic review and meta-analysis is to rigorously evaluate the associations between esports participation and multidimensional health indicators. Specifically, this study aims to: (1) quantitatively compare the differences in health outcomes between esports players and non-esports players; and (2) explore the correlations between the level of esports engagement and various health indicators. Additionally, we seek to systematically assess study quality and synthesize existing findings to provide a comprehensive overview of the current evidence. Furthermore, by stratifying participation levels and applying clear operational definitions, we intend to investigate potential differences in health outcomes between professional and non-professional players, thereby generating more targeted and nuanced findings.

By achieving these objectives, this synthesis will contribute to the preliminary evidence-based foundations for industry development and health promotion strategies across participation levels while identifying specific risk factors and protective mechanisms. These findings will be particularly crucial as esports continues its integration into mainstream competitive frameworks, helping to inform its developmental trajectory and support systems for participants at all levels. However, given the emerging nature of this field and the aforementioned methodological heterogeneity across existing literature, our synthesis is intended to be tentative and exploratory, rather than drawing definitive conclusions.

## Methods

The systematic review was conducted following the Preferred Reporting Items for Systematic Reviews and Meta-Analyses (PRISMA) guidelines [43]. The PRISMA checklist is provided in S1 Supplementary Information 1. The review protocol was prospectively registered in the International Prospective Register of Systematic Reviews (PROSPERO) database (Registration ID: CRD42024622812).

### Search Strategy

Literature searches were performed in PubMed, Web of Science, Scopus, SPORTDiscus, MEDLINE, Esports Research Network, and ProQuest from inception to May 13, 2026. The Esports Research Network database was specifically searched for esports-specific literature, while ProQuest was utilized to capture gray literature. The search strategy was built upon two main conceptual domains: (1) esports and competitive gaming terminology (e.g., “esports”, “cyber sports”, “professional gaming”, specific game genres like “MOBA” or “FPS”, and popular game titles such as “League of Legends” and “Counter-Strike”); and (2) health-related outcomes (e.g., “health”, “injury”, “fitness”, “physical activity”, “sleep”, “anxiety” and “depression”). Boolean operators (AND, OR, NOT) and truncation symbols (e.g., “*”) were utilized to combine these domains, capture word variations, and exclude irrelevant terms (e.g., “esporte”, “gamma”). The complete search strategy, including the exact search strings, filters, and results for each database, is available in S1 Supplementary Information 2. Additionally, the reference lists of all included articles and relevant previous systematic reviews or meta-analyses [1, 3, 18, 22, 32, 41, 44–46] were hand-searched to identify potentially eligible studies that might have been missed through database searching. All retrieved citations were managed using EndNote 20.

### Study Selection

Following automatic and manual deduplication, two independent reviewers (DT and XZ) screened titles and abstracts for initial eligibility. Full texts of potentially relevant articles were then assessed against the inclusion criteria. The calculated kappa value was 0.81, indicating excellent agreement between the reviewers. Any discrepancies between reviewers were resolved by consultation with a third reviewer (JL). When full texts or complete data were unavailable, corresponding authors were contacted.

### Eligibility Criteria

The following inclusion and exclusion criteria were established a priori. Studies were included if they: (a) identified participants as esports players, with all esports modalities and levels being considered; and (b) investigated at least one health-related outcome in esports players by either comparing health indicators between esports players (EP) and non-esports players (NP) or analyzing correlations between esports participation parameters (frequency, duration, daily/weekly gaming hours) and health-related outcomes. To capture a comprehensive range of evidence, health was conceptualized broadly based on a modern adaptation of the World Health Organization (WHO) definition, which emphasizes “the ability to adapt and to self-manage” [47]. Consequently, eligible outcomes broadly encompassed any indicators related to physical functioning, mental well-being, social connectivity, and health-related behaviors.

Studies were excluded if they: (a) focused solely on general video game players; (b) were not publications in English or Chinese; (c) presented incomplete data or had inaccessible full texts; or (d) were qualitative studies, review articles (narrative reviews, scoping reviews, systematic reviews), consensus papers, or opinion articles (letters to the editor, viewpoints, comments). The language restriction to English and Chinese was applied because English is the primary language for international scientific publications, and the combination of both languages covers the vast majority of core literature globally. Other languages were excluded due to the research team’s language proficiency and translation feasibility, which was necessary to ensure strict accuracy during data extraction and quality assessment. The selection process did not impose restrictions based on participants’ demographic characteristics such as age, sex, ethnicity, or geographical location.

To standardize the inclusion criteria and address the currently ambiguous definitions of esports in primary studies, we adopted a more comprehensive definition proposed by Pedraza-Ramirez et al. as our foundational framework [48]. This definition provides detailed criteria encompassing multiple skill levels, platforms, and formats, which align with the broad scope of esports examined in this review and enhance our methodological transparency.

Furthermore, “game characteristics” served as a crucial auxiliary method. Specifically, if a study reported that players participated in fair, competition-centered adversarial games where individual skills and strategies are key to winning, or current mainstream esports titles, or if it exclusively investigated players of a specific mainstream esports game, the study was included even if the authors broadly referred to the subjects as “gamers or video game players” rather than explicitly defining them as “esports players”. Conversely, studies that explicitly used the term “esports players” but reported predominantly non-competitive game genres (e.g., virtual world exploration, role-playing games, or simulation/nurturing games) were strictly excluded. Additionally, we adopted a broad definition of esports encompassing various skill levels, where “competition” denotes the inherent competitive nature within the game itself, rather than only being strictly defined by externally organized tournaments.

### Data Extraction and Quality Assessment

Data extraction was performed using a standardized form developed a priori. Two reviewers (DT and XZ), not blinded to the study authors, independently extracted data from all included studies and assessed the quality of the included studies. Any disagreements were resolved through discussion, and if necessary, by consulting a third reviewer (JL). The extracted data comprised study characteristics (first author, year of publication, study design), population characteristics (sample size, age, sex distribution, country), esports-specific information (game genre, professional level), and outcome measures. For outcome variables intended for quantitative synthesis, numerical data were collected directly from the main text and tables based on the specific analysis type. Specifically, for studies comparing esports and non-esports groups, we extracted the sample sizes, means, and standard deviations (SDs) of baseline health outcomes. For studies investigating the association between esports engagement and health indicators, we extracted the reported correlation coefficients. The most recent or complete version of each study was selected as the primary reference. In cases of incomplete data reporting, we attempted to contact the corresponding authors via email to request the missing information. However, we only contacted authors if the manuscript explicitly indicated that the relevant health outcomes were measured or analyzed. We did not solicit data from authors of general esports health studies if there was no indication that the specific variables of interest were assessed.

The methodological quality and risk of bias were assessed using the Joanna Briggs Institute (JBI) critical appraisal checklist, modified for cross-sectional studies. Although a small proportion of the included studies featured longitudinal or experimental designs, our analysis exclusively utilized their baseline cross-sectional data. In accordance with the guidelines in the Cochrane Handbook [49], which emphasize that the risk of bias should be assessed for the specific result being analyzed rather than the overall study design, we evaluated these studies based solely on the cross-sectional data extracted. Evaluating them with longitudinal tools would inappropriately downgrade their quality based on domains irrelevant to baseline data.

### Data Synthesis and Analysis

All statistical analyses were performed using Review Manager V.5.3 software developed by the Cochrane Collaboration. For the quantitative synthesis, we employed different approaches based on the nature of the outcome data. For comparisons between esports and non-esports groups, mean differences (MD) were calculated when outcomes were measured on the same scale across studies. When different measurement scales were used, standardized mean differences (SMD) with 95% confidence intervals (CI) were computed. For correlation studies examining the relationship between esports participation and health indicators, correlation coefficients (r) were transformed to Fisher’s Z scores to achieve approximate normality of the sampling distribution and subsequently back-transformed to correlation coefficients for the presentation and interpretation of pooled results. When correlation coefficients were not directly reported, we converted other effect size measures to correlation coefficients using formulas recommended by the Cochrane Handbook for Systematic Reviews [49].

Statistical heterogeneity was quantified using the I^2^ statistic and the Q test. The selection of the meta-analytical model was primarily guided by the clinical and methodological diversity of the included studies, rather than solely relying on statistical heterogeneity. Given the inherent variations in esports populations (e.g., game genres, levels, sex) and study designs, a random-effects model was primarily utilized.

To ensure robust synthesis, we conducted a leave-one-out sensitivity analysis to assess the impact of each study on the overall pooled estimate. Subgroup analyses were performed when sufficient data were available, stratifying by professional level. Specifically, participants were classified as “professional” if the original studies explicitly reported that they were recruited from registered esports clubs or organizations, participated in official top-tier tournaments, or self-reported esports as their primary profession (i.e., playing for a living). Conversely, participants were classified as “non-professional” if they were recruited from university esports clubs, general gaming communities, or were explicitly identified as recreational players participating in esports primarily for leisure. Due to the limited number of available studies and the inconsistent reporting standards and the incomplete information across the original literature, we did not further subdivide these groups into more granular categories. Publication bias was visually assessed using funnel plots for all synthesized outcomes. In accordance with the recommendations of the Cochrane Handbook [49], formal evaluation and interpretation of publication bias in the main text were restricted to outcomes containing 10 or more studies. For outcomes comprising fewer than 10 studies, where statistical power is limited, the generated funnel plots are provided in Supplementary Material 1 for reference and to ensure full reporting transparency.

Meta-analyses were conducted only when at least two studies assessed the same outcome and provided sufficient data. Otherwise, a narrative synthesis was performed. When substantial heterogeneity was present but limited study numbers precluded further exploration of its sources, pooled estimates were treated as exploratory analyses rather than definitive conclusions and should be interpreted with caution.

## Results

### Search Results

**Fig. 2:**
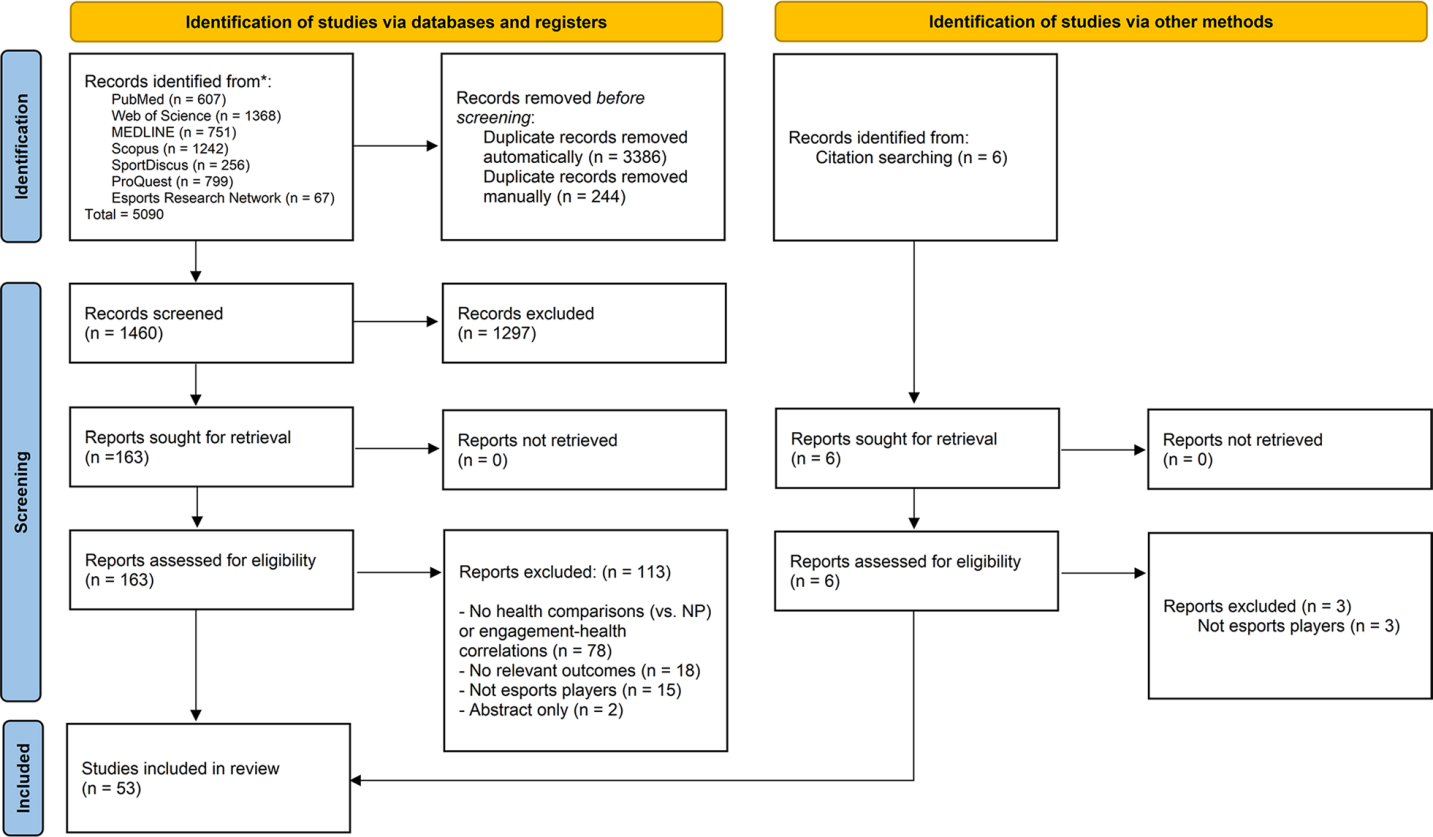
PRISMA flow diagram for study selection. *NP* Non-esports players

The comprehensive literature search retrieved 5090 potentially relevant articles. Following the application of inclusion and exclusion criteria, 53 studies were included in the systematic review, among which 36 studies provided appropriate data for meta-analysis. A PRISMA flow diagram detailing the study selection process is shown in Fig. 2.

### Study Characteristics

A systematic review identified 53 studies published between 2012 and 2026, with sample sizes ranging from 19 to 70,145 participants. The methodological approaches included 1 mixed-methods study, 3 interventional studies, and 49 observational studies. The cumulative sample comprised 74,891 participants in comparative studies between EP and NP (n = 23 studies), while 11,761 participants were included in correlational studies examining associations between esports participation and health indicators (n = 30 studies). The studies represented diverse geographical regions, encompassing Asian countries (China, Thailand, South Korea, Japan, Indonesia, and Saudi Arabia), North America (the United States and Canada), South America (Brazil), European nations (Turkey, Germany, Poland, Italy, Spain, Norway, Russia, Ukraine, Denmark, and Switzerland), and Oceania (Australia). Participants primarily engaged in various esports genres, including First-Person Shooter (FPS), Fighting Games (FG), Multiplayer Online Battle Arena (MOBA), Real-Time Strategy (RTS), Sports Video Games (SVG), and Strategy Card Games (SCG). The detailed characteristics of the included studies can be found in Table 1. The health-related outcomes identified in these studies were systematically categorized into four major domains: behavioral factors, physical health, mental health, and social health (including social connectivity and adaptive functioning). Additionally, several cross-cutting measures were identified, including general health status, perceived health, and quality of life indicators. This classification framework, adapted from categorizations proposed in previous scoping reviews on the esports health research landscape and aligned with the WHO’s definition of health [22, 47], provides a comprehensive structure for understanding the multifaceted relationship between esports participation and health outcomes, as detailed in Table 2.

**Table 1:** Characteristics of the included studies

Study		Design	Country	Sample (n, male)	Age (SD)	Genre	Comparison	Level	Relevant outcomes
Han et al. 2012 [52]^a^		Observational	Korea	55 (NR)	EP: 20.8 (1.5) NP: 20.9 (2.1)	NR	NR	Pro	Smoking, drinking, depression
Hyun et al. 2013 [108]		Observational	Korea	23 (23)	19.8 (1.7)	RTS	/	Pro	Cortical thickness, cognitive flexibility
Adachi and Willoughby 2016 [121]		Observational	Canada	1132 (333)	19.08 (NR)	FPS, FG, AVG	/	Non-Pro	Gaming frequency and sports participation
Goh et al. 2019 [73]^b^		Observational	Multi	149 (142)	Over 18	MOBA	/	Non-Pro	Gaming hours and psychological well-being
Aggarwal et al. 2020 [72]^b^		Observational	Multi	44 (42)	21.7 (NR)	FPS	/	Non-Pro	Rounds played and ADHD symptoms, gaming disorders, anxiety
Bayrakdar et al. 2020 [14]^b^		Observational	Multi	137 (137)	19.92 (2.21)	NR	/	Pro	Gaming hours and PA, BMI
Kang et al. 2020 [66]^a^		Observational	Korea	172 (NR)	EP: 21.3 (1.4) NP: 21.3 (1.5)	FPS, MOBA, RTS	NR	Pro	Anxiety
Lee et al. 2020 [50]^a^		Mixed	Korea	54 (54)	EP: 20.41 (2.40) NP: 21.95 (2.92)	MOBA, FPS	NR	Pro	BMI, caffeine consumption, sleep, anxiety, depression, stress
Rudolf et al. 2020 [13]^b^		Observational	Germany	1066 (980)	22.9 (5.9)	FPS, MOBA, RTS, SVG	/	Pro & Non-Pro	Gaming hours and sedentary behavior, BMI, perceived health
Trotter et al. 2020 [12]		Observational	Multi	1772 (NR)	NR	NR	/	Non-Pro	Gaming frequency/rank and drinking, smoking, general health, PA, perceived PA
Giakoni-Ramírez et al. 2021 [23]^b^		Observational	Spain	53 (NR)	21.01 (0.39)	MOBA, FPS, SCG, SVG	/	Pro	Gaming experience and body composition
Kelly et al. 2021 [75]		Observational	Australia	905 (348)	NR	NR	/	Non-Pro	Gaming frequency and life satisfaction, social connection, physical activity, soft drinks, alcohol consumption, smoking, sleep
Luzio et al. 2021 [122]		Interventional	Italy	42 (31)	25.2 (4.63)	FPS	NG	Non-Pro	Gaming frequency and multisensory temporal precision, migraine incidence
Madden and Harteveld 2021 [123]^b^		Observational	Multi	68 (53)	22.9 (3.36)	FPS, MOBA mainly	/	Non-Pro	Gaming hours and sleep duration, PA, health status, mental health, stress
Mendoza et al. 2021 [69]		Observational	Spain	45 (45)	18–27	MOBA	NR	Pro & Non-Pro	Pre-competition anxiety
DiFrancisco-Donoghue et al. 2022 [20]^a^		Observational	US	24 (24)	EP: 20.2 (1.7) NP: 19.2 (1.3)	MOBA, FPS	NG	Non-Pro	BMI, sleep, PA, body composition,
Giakoni-Ramírez et al. 2022 [124]^b^		Observational	Multi	260 (260)	21.3 (2.26)	MOBA, FPS, SCG	/	Pro	Gaming experience and PA, weight, sedentary behavior
Harris et al. 2022 [68]		Observational	NR	688 (510)	25.2 (6.2)	FPS	/	Non-Pro	Gaming frequency and gaming disorders
Lam et al. 2022 [19]^b^		Observational	China	50 (50)	20.0 (1.67)	MOBA	/	Pro	Gaming experience and body composition, total number of body regions with health problems
Luo et al. 2022 [70]		Observational	China	63 (46)	19.65 (1.05)	MOBA, FPS	/	Non-Pro	Gaming hours and mental fatigue
Ohno 2022 [125]^b^		Observational	Japan	874 (448)	18.5 (2.13)	MOBA, SVG, Racing	/	Non-Pro	Gaming hours and online gaming addiction, sense of underachievement, aggressive feelings
Soares et al. 2022 [34]^a^		Observational	Brazil	401 (213)	EP: 22.49 (3.31) NP: 24.5 (6.35)	NR	NR	Non-Pro	Depression, stress, anxiety, social connection
Trotter et al. 2022 [76]^a^		Observational	Australia	188 (120)	Range: 13 to 18 EP: 14.28 NP: 14.57	NR	NR	Non-Pro	Self-regulation, growth mindset, positive youth development, PA, sports participation, general health
Basuodan et al. 2023 [62]		Observational	Saudi Arabia	198 (115)	24.7 (4.2)	NR	NR	Pro	Gaming hours/experience and carpal tunnel syndrome
Cheng et al. 2023 [36]^b^		Observational	China	1441 (758)	NR (college students)	NR	/	Non-Pro	Gaming experience/hours/frequency and sleep, mobile phone addiction, online game addiction, life satisfaction, perceived stress
Cueva-Reguera et al. 2023 [61]^a^		Observational	Spain	20 (NR)	EP: 21.90 (1.19) NP: 22.30 (1.25)	NR	NR	Pro	BMI, trunk stabilizer muscle
Ketelhut et al. 2023 [9]^a^		Observational	Switzerland	102 (98)	EP: 23 (3) NP: 24 (3)	MOBA, FPS, SVG	NG	Pro	BMI, body fat, blood pressure, pulse wave velocity, VO_2max_, grip strength, PA
Kulecka et al. 2023 [57]^a^		Observational	Poland	141 (141)	EP: 20.88 (2.37) NP: 22.89 (2.15)	NR	NR	Pro	BMI, PA, body composition, gut microbiome
Luo et al. 2023 [74]		Observational	China	216 (154)	19.71 (1.56)	MOBA mainly	/	Non-Pro	Gaming hours and loneliness
Mancı and Özdalyan 2023 [126]^b^		Observational	Turkey	158 (125)	19.81 (1.88)	NR	/	Pro & Non-Pro	Gaming experience/hour and PA
Ren and Nie 2023 [127]^a^		Interventional	China	180 (NR)	22.85 (1.26)	MOBA, FPS	NR	Non-Pro	BMI
Study		Design	Country	Sample (n, male)	Age (SD)	Genre	Comparison	Level	Relevant outcomes
Shan et al. 2023 [128]^b^		Observational	China	453 (192)	21.5 (1.9)	MOBA, FPS	/	Non-Pro	Gaming hours and psychological well-being
Soffner et al. 2023 [129]^b^		Observational	Germany	817 (703)	24.2 (6.9)	NR	NR	Pro & Non-Pro	Gaming hours and well-being, PA, sedentary time, health status, drinking, dietary behavior, BMI
Tang et al. 2023 [21]^a^		Observational	China	1549 (632)	EP: 18.8 (1.5) NP: 18.6 (1.5)	FPS, MOBA, SCG, SVG, RTS, FG	NR	Non-Pro	Sports participation, sleep, musculoskeletal pain, hearing, eye/vision, malnutrition
Uluağaç et al. 2023 [63]		Observational	Turkey	20 (20)	20.85 (2.32)	MOBA	/	Pro	Gaming experience/hours and hand function, strength, coordination, pain
Arslan et al. 2024 [53]		Observational	Turkey	248 (204)	22.19 (5.97)	NR	/	Pro & Non-Pro	Gaming hours and night eating syndrome, food addiction
Bäcklund et al. 2024 [130]^b^		Observational	Multi	321 (303)	23.25 (4.51)	MOBA	/	Non-Pro	Gaming hours and online game addiction
Chaiwiang and Koo-Akarakul 2024 [51]^a^		Observational	Thailand	160 (160)	EP: 28.48 (4.26) NP: 26.95 (4.65)	NR	NR	Pro	Depression, anxiety, stress, computer vision syndrome, ophthalmic examination, alcohol consumption, smoke
Cyma-Wejchenig et al. 2024 [35]^a^		Observational	Poland	61 (42)	EP: 22.92 (2.7) NP: 22.32 (2.23)	NR	NG	Non-Pro	BMI, musculoskeletal pain, perceived health, PA
Kowalski et al. 2024 [65]		Observational	Poland	166 (104)	EP: 21.68 (2.2) NP: 21.84 (2.8)	NR	NR	Non-Pro	Body surface area, respiratory muscle function
Mancı et al. 2024 [131]^a^		Interventional	Turkey	19 (19)	EP: 27.40 (3.09) NP: 26.55 (5.05)	FPS mainly	NG	Non-Pro	BMI, PA
Monma et al. 2024 [60]		Observational	Japan	175 (79)	21.6 (5.6)	FPS, MOBA	/	Pro & Non-Pro	Gaming hours/experience and vision, headache, musculoskeletal pain
Overå et al. 2024 [132]^a^		Observational	Norway	70,145 (34,572)	16–18	NR	NR	Non-Pro	General health, loneliness, well-being, depression, PA
Pradnyadewi et al. 2024 [95]		Observational	Indonesia	150 (147)	15.3 (1.27)	MOBA	/	Non-Pro	Gaming hours and De Quervain syndrome
DiFrancisco-Donoghue et al. 2025 [58]^a^		Observational	US	60 (30)	EP:20.9 (3.12) NP:21.95 (3.29)	MOBA, FPS, SVG	NG	Non-Pro	BMI, body composition, grip strength, sedentary behavior, musculoskeletal pain
Kidcaff et al. 2025 [133]^b^		Observational	Multi	243 (198)	21.27 (4.04)	MOBA, FPS, FG, SVG	/	Non-Pro	Gaming hours and sleep quality
Onishchenko et al. 2025 [59]^a^		Observational	Russia	52 (52)	14.9 (1.63)	NR	/	Non-Pro	BMI, body composition
Sand Hansen et al. 2025 [134]^a^		Observational	Denmark	251 (163)	EP:19.1 (2.9) NP:17.2 (2.2)	FPS, MOBA	NR	Non-Pro	BMI
Solmaz et al. 2025 [67]^b^		Observational	Turkey	403 (288)	19.01 (2.86)	MOBA, FPS	/	Non-Pro	Gaming hours and gaming disorders, anxiety, sleep
Birol et al. 2026 [64]		Observational	Turkey	87 (79)	23.78 (3.33)	NR	/	Pro & Non-Pro	Gaming hours and voice problems
Luts 2026 [135]^a^		Observational	Ukraine	41 (41)	17–25	NR	NR	Non-Pro	BMI, body composition
Şarahman Kahraman et al. 2026 [136]^b^		Observational	Turkey	235 (201)	23.9 (4.4)	NR	/	Non-Pro	Gaming hours and stress
Yamamoto et al. 2026 [71]		Observational	Japan	275 (269)	24.8 (11.8)	FPS, SCG, SVG	/	Pro & Non-Pro	Esports training hours and psychological distress

*ACG* Action Video Game; *ADHD* Attention Deficit Hyperactivity Disorder; *BMI* Body Mass Index; *EP* Esports Players; *FG* Fighting Games; *FPS* First-Person Shooter; *MOBA* Multiplayer Online Battle Arena; *NG* Non-Video Gamers; *NP* Non-Esports Players; *NR* Not Reported; *PA* Physical Activity; *Pro* Professional; *RTS* Real-Time Strategy Games; *SCG* Strategy Card Games; *SVG* Sports Video Games

^a^Studies included in the meta-analysis comparing health outcomes between esports players and non-esports players

^b^Studies included in the meta-analysis analyzing correlations between esports participation and health outcomes

**Table 2:** Classification of health-related outcomes

Categories	Sub-categories	Outcomes	n	Study
Behavior factors	Physical activity	Sports participation, physical activity level, and sedentary behavior	18	[9, 12–14, 20, 21, 35, 57, 58, 75, 76, 121, 123, 124, 126, 129, 131, 132]
	Lifestyle habits	Diet/nutrition (food addiction, night eating syndrome, eating behavior, malnutrition, dietary behavior), sleep (sleep pattern, sleep quality, insomnia), and substance use (smoking, alcohol consumption, caffeine consumption)	12	[12, 20, 21, 36, 50–53, 75, 123, 129, 133]
Physical health	Anthropometric measures	Body mass index, body fat, body surface area, muscle mass, body water, lean body mass, bone mass	17	[9, 13, 14, 19, 20, 23, 50, 57–59, 61, 124, 127, 129, 131, 134, 135]
	Musculoskeletal system	Pain/discomfort, muscle function/strength, trunk stabilizer muscle, and other specific conditions (De Quervain syndrome, carpal tunnel syndrome)	9	[9, 19, 21, 35, 58, 60, 62, 63, 95]
	Cardiorespiratory function and other physiological systems	Brain structure, cortisol, sensorimotor response, blood pressure, pulse wave velocity, respiratory function, maximal oxygen consumption, gut microbiome, vision issues, hearing function, headache/migraine, and voice problems	9	[21, 51, 57, 58, 60, 64, 65, 108, 122]
Mental health	Clinical aspects	Depression, anxiety, stress, gaming disorder, ADHD symptoms, psychological distress, and burnout	18	[34, 36, 50–53, 66–72, 123, 125, 130, 132, 136]
	Psychological well-being	Life satisfaction, self-regulation, growth mindset, loneliness, and psychological well-being	11	[36, 73–76, 108, 123, 125, 128, 129, 132]
Social health	Social health	Social connection, social adaptation, positive youth development	3	[34, 75, 76]

A study may be counted in more than one sub-category when it reports outcomes spanning multiple sub-categories

### Risk of Bias

The overall appraisal score across the 36 studies included in the meta-analysis was 64.2%, reflecting a moderate risk of bias. Of these, 6 studies were classified as high quality (≥ 80%), 24 studies as moderate quality (50%–79%), and 6 studies as low quality (< 50%).

**Fig. 3:**
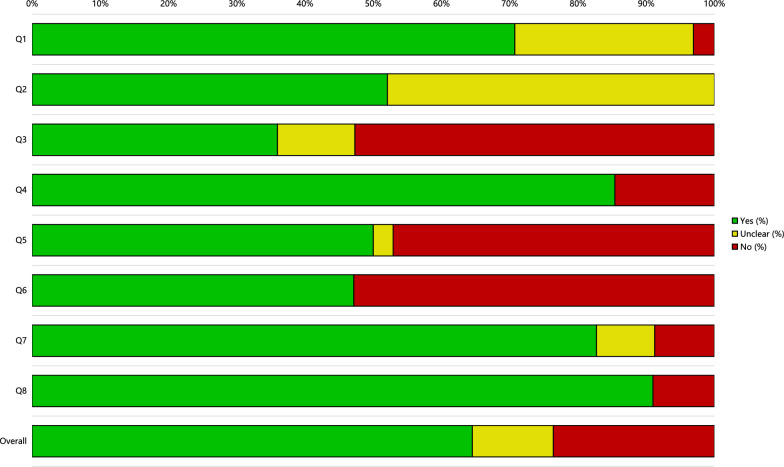
Methodological quality assessment of the included studies. Q1: Were the criteria for inclusion in the sample clearly defined? Q2: Were the study subjects and the setting described in detail? Q3: Was the exposure measured in a valid and reliable way? Q4: Were objective, standard criteria used for measurement of the condition? Q5: Were confounding factors identified? Q6: Were strategies to deal with confounding factors stated? Q7: Were the outcomes measured in a valid and reliable way? Q8: Was appropriate statistical analysis used?

The highest compliance rates were observed for appropriate statistical analysis (88.9%), while the lowest were for the validity of exposure measurement and the control of confounding factors (36.1% and 47.2%, respectively). Figure 3 illustrates the overall scores of the included studies across various domains. This variability highlights methodological limitations, particularly in study design, reporting, and exposure measurement reliability. Moreover, the high risk of bias exhibited in the domains of “subject criteria” and “exposure measurement” further reflects the prevailing ambiguity in the current definitions of esports and esports players. This also exposes a critical issue: the current assessment of esports participation lacks objective and unified standards. Consequently, the moderate overall quality of the included studies compromises the certainty of the current evidence base, indicating that the meta-synthesized results should be interpreted with caution. Detailed quality assessment results for each study are provided in S1 Table 1.

### Meta-Analysis

The meta-analytic procedures were conducted across two distinct analytical frameworks. First, comparative analyses between EP and NP encompassed 8 health parameters (n = 19 studies): body mass index (BMI), physical activity (PA), depression, anxiety, stress, sleep duration, body fat percentage, and grip strength. Second, correlational analyses examining associations between esports participation parameters (frequency, duration, intensity, and daily/weekly gaming hours) and health outcomes included seven indicators (n = 17 studies): BMI, psychological well-being, gaming disorders, stress, PA, sedentary behavior, and sleep quality.

#### Physical Activity

**Fig. 4:**
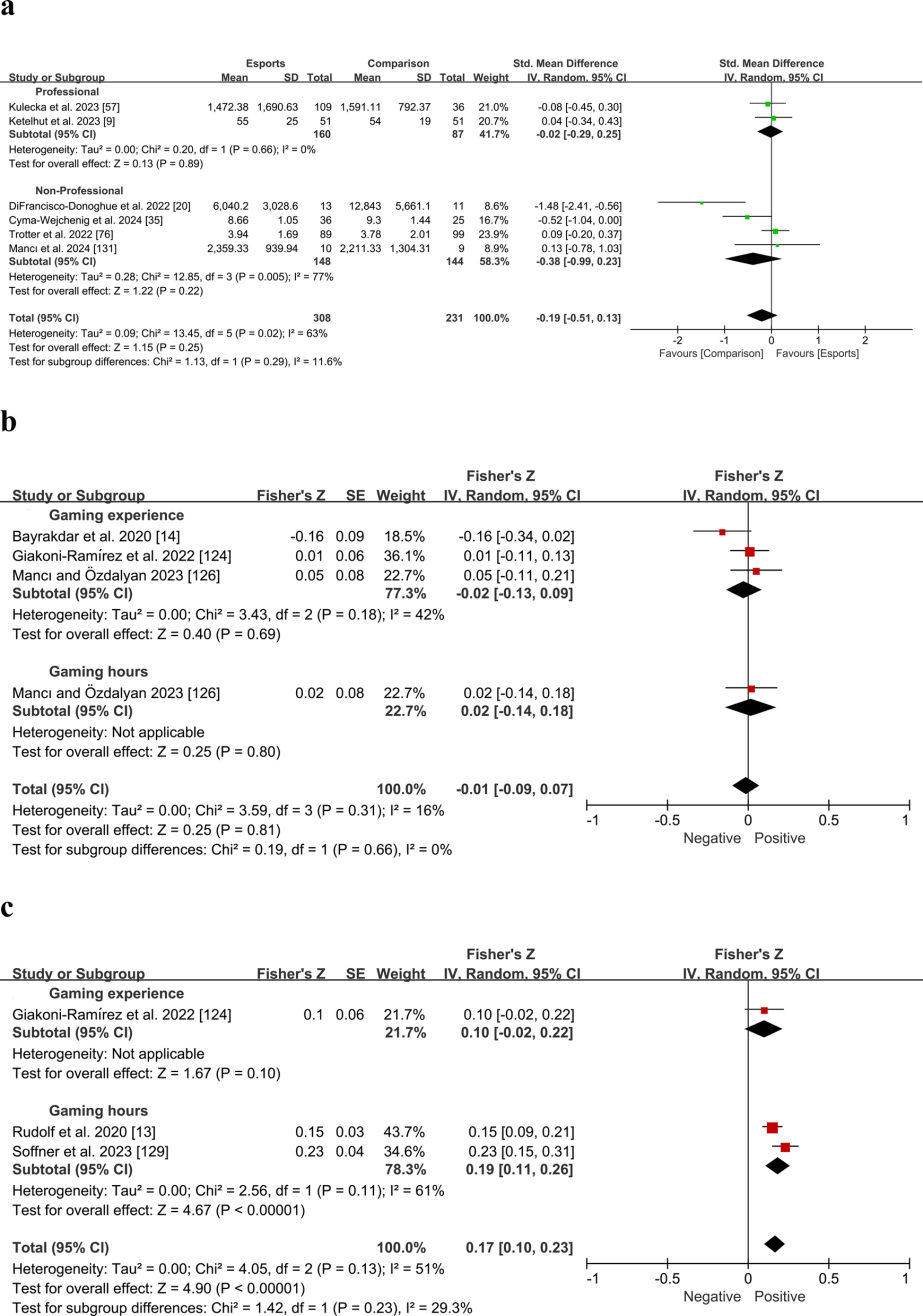
Forest plots of physical activity and sedentary behavior. **a** Differences in physical activity between esports players and non-esports players; **b** correlation between esports participation and physical activity; and **c** correlation between esports participation and sedentary behavior. *CI* Confidence Interval, *df* Degrees of Freedom, *IV* Inverse Variance, *SD* Standard deviation, *Std*. Standardized

The comparison of physical activity levels between EP and NP was examined across six studies (n = 539; 308 EP, 231 NP). The initial pooled analysis showed no significant difference between groups (SMD –0.19, 95% CI –0.51 to 0.13, *p* = 0.25). Substantial heterogeneity was observed in the initial analysis (*I*^*2*^ = 63%, *p* = 0.02) (Fig. 4a).

Subgroup analysis comparing professional and non-professional players revealed no significant subgroup differences. However, it is noteworthy that no substantial heterogeneity was observed within the professional subgroup (*I*^*2*^ = 0). Furthermore, a leave-one-out sensitivity analysis demonstrated that excluding the study by DiFrancisco-Donoghue et al. [20], which reported the most extreme effect size (SMD –1.48, 95% CI –2.41 to –0.56), substantially reduced the overall heterogeneity (*I*^*2*^ decreased from 63 to 7%). Upon further examination, the study by DiFrancisco-Donoghue et al. [20]. had a substantially smaller sample size (n = 24) compared to the majority of studies included in the analysis. Additionally, significant baseline differences in physical characteristics were observed between the two groups in their study. These factors likely contributed to its divergence from the other included studies. Future research should further explore these sources of heterogeneity as more high-quality studies become available. However, following this exclusion, the pooled estimate remained non-significant (SMD –0.04, 95% CI –0.23 to 0.15, *p* = 0.71). The complete results of the sensitivity analysis are provided in S1 Table 3.

Four studies examined the correlation between esports participation and physical activity levels. The overall pooled analysis revealed no significant correlation (r = –0.01, 95% CI –0.09 to 0.07) (Fig. 4b). Subgroup analyses based on the measurement of esports participation showed no significant subgroup differences. Neither gaming experience nor gaming hours were significantly correlated with physical activity.

Regarding sedentary behavior, the pooled analysis of three studies demonstrated a small but significant positive correlation with esports participation (r = 0.17, 95% CI 0.10 to 0.23, *p* < 0.001), with moderate heterogeneity (*I*^*2*^ = 51%, *p* = 0.13) (Fig. 4c). In the subgroup analysis, gaming hours showed a significant positive correlation (r = 0.19, 95% CI 0.11 to 0.25, *p* < 0.001), whereas the single study assessing gaming experience indicated a non-significant positive trend (r = 0.10, 95% CI –0.02 to 0.22, *p* = 0.10).

#### Lifestyle Habits

**Fig. 5:**
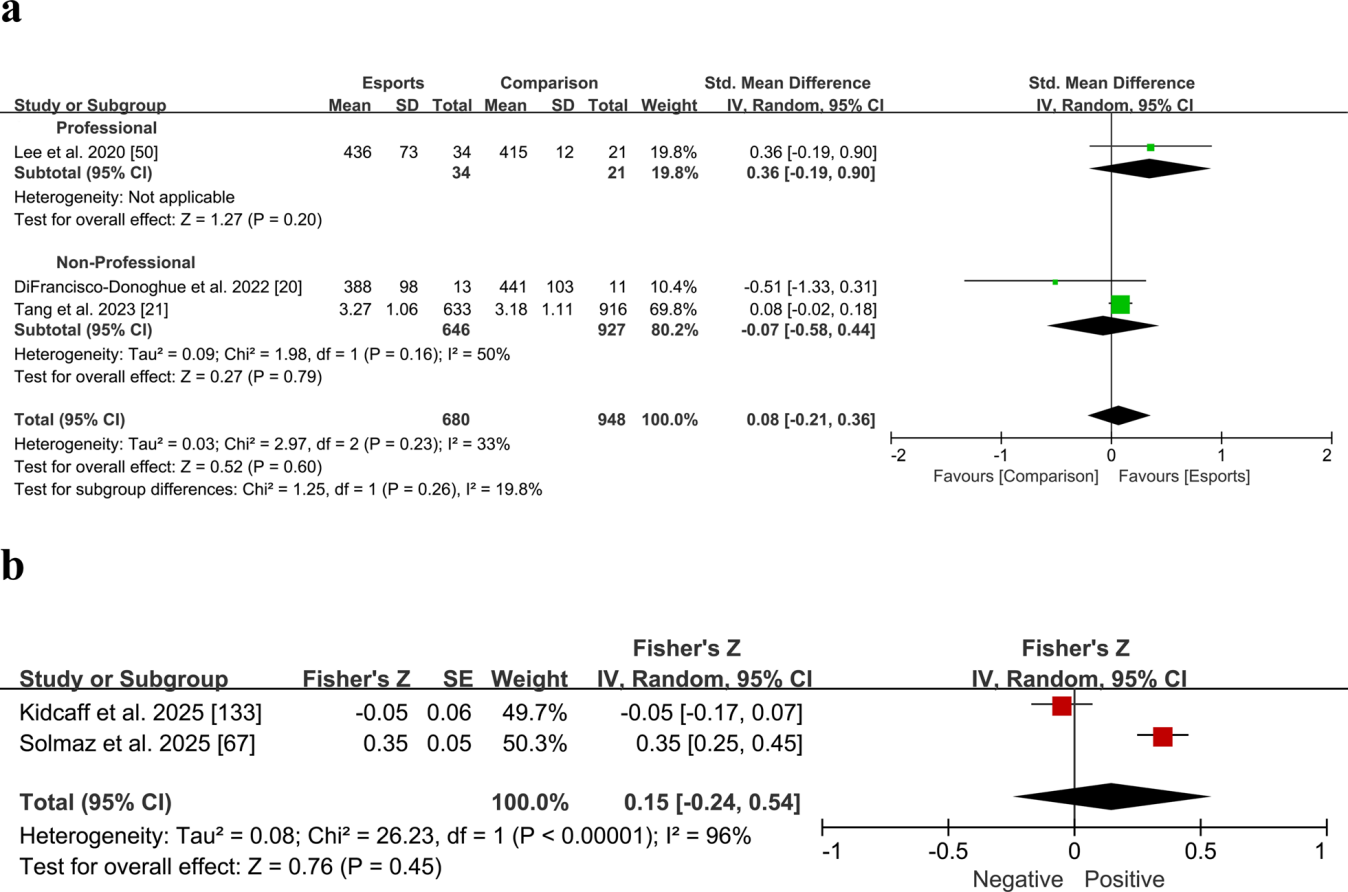
Forest plots of sleep outcomes. **a** Differences in sleep duration between esports players and non-esports players; and **b** correlation between esports participation and sleep quality. *CI* Confidence Interval, *df* Degrees of Freedom, *IV* Inverse Variance, *SD* Standard deviation, *Std.* Standardized

The comparison of sleep duration between EP and NP was examined across three studies (n = 1,628; 680 EP, 948 NP). The pooled analysis showed no significant difference between groups (SMD 0.08, 95% CI –0.21 to 0.36; *p* = 0.23), and subgroup analyses revealed no significant between-group differences (Fig. 5a). Additionally, current evidence suggests a non-significant correlation between gaming hours and sleep quality (r = 0.15, 95% CI –0.24 to 0.49, *p* = 0.45) (Fig. 5b).

Given the limited number of eligible studies, quantitative synthesis of several lifestyle variables was precluded. Thus, a narrative synthesis is provided. Unlike the quantitatively pooled results, these narrative summaries lack statistical robustness and should not be construed as providing definitive conclusions. Therefore, they warrant cautious interpretation. No significant differences were observed in the prevalence of malnutrition (EP: 4.4%, NP: 3.5%, *p* = 0.555) [21] or caffeine consumption (EP: 61.8%, NP: 54.2%, *p* = 0.536) [50]. Alcohol consumption patterns showed inconsistent results across studies. Chaiwiang et al. reported significantly higher alcohol consumption in EP (63.16% vs 44.59%, *p* = 0.023) [51], while Han et al. found no significant difference (47.1% vs 50.0%, *p* = 0.862) [52]. In terms of smoking habits, however, both studies found no significant differences between groups (EP: 23.5% vs NP: 22.2%, *p* = 0.927; EP: 12.16% vs NP: 10.53%, *p* = 0.754) [51, 52].

Additionally, Arslan et al. reported that each additional hour of weekly esports engagement was associated with increased risks of both food addiction (OR: 1.022, *p* < 0.05) and night eating syndrome (OR: 1.034, *p* < 0.05) [53]. Regarding sleep quality, Lee et al. found no significant difference in insomnia severity between EP and NP (8.97 ± 4.13 vs 8.05 ± 5.27, *p* = 0.508) [50].

#### Anthropometric Measures

**Fig. 6:**
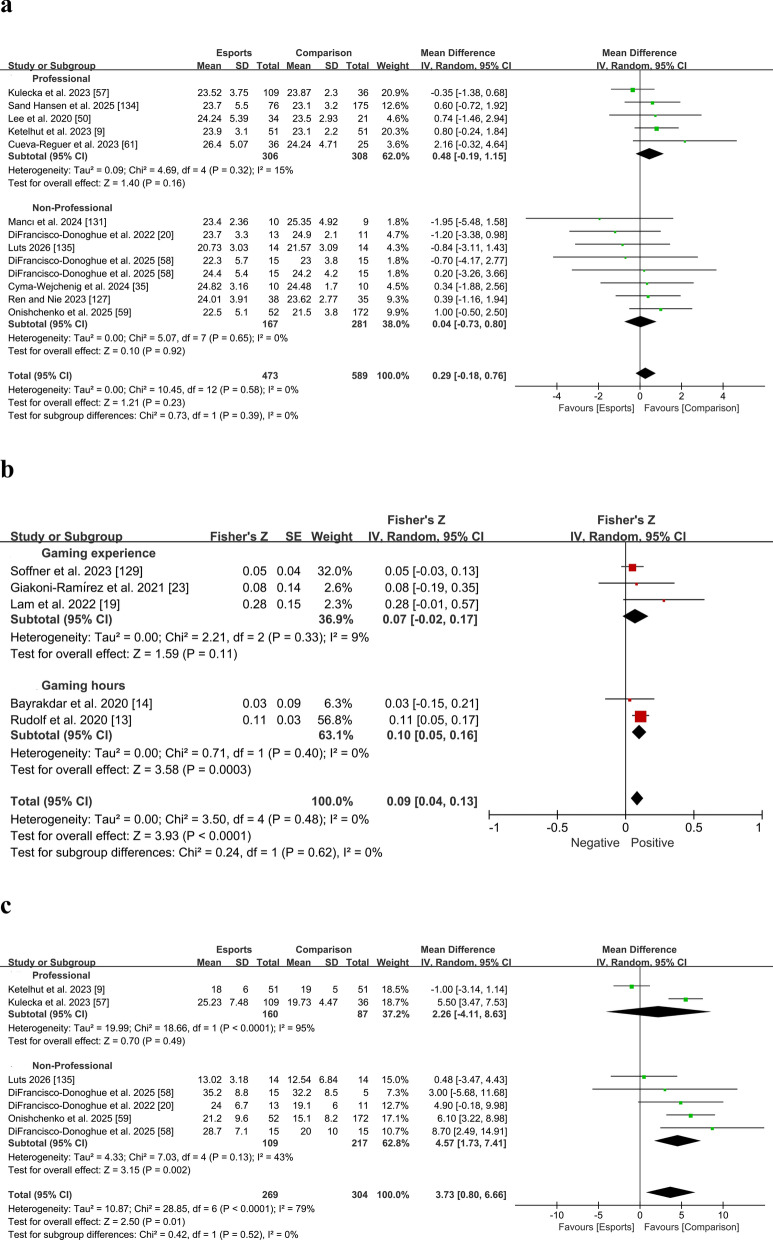
Forest plots of anthropometric measures. **a** Differences in body mass index between esports players and non-esports players; **b** correlation between esports participation and body mass index; and **c** differences in body fat percentage between esports players and non-esports players. *CI* Confidence Interval, *df* Degrees of Freedom, *IV* Inverse Variance, *SD* Standard deviation, *Std.* Standardized

The analysis of BMI between EP and NP included 12 studies (n = 1,062; 473 EP, 589 NP). The pooled analysis revealed no significant difference between EP and NP (MD 0.29, 95% CI –0.18 to 0.80, *p* = 0.23), with negligible between-study heterogeneity (*I*^*2*^ = 0%, *p* = 0.58). The test for subgroup differences indicated no significant variation between professional and non-professional levels (Fig. 6a). From a clinical perspective, a mean difference of 0.29 kg/m^2^ in BMI is negligible and does not translate to a meaningful shift in weight categories or associated cardiovascular risks [54]. Regarding the correlation between esports participation and BMI, our meta-analysis included five studies, categorized into gaming experience (3 studies) and gaming hours (2 studies). The overall pooled analysis demonstrated a significant positive correlation (r = 0.09, 95% CI 0.04 to 0.13; *p* < 0.001) (Fig. 6b). Although the test for subgroup differences was not statistically significant, the gaming experience subgroup showed a non-significant correlation (r = 0.07, 95% CI –0.02 to 0.17, *p* = 0.11), whereas the gaming hours subgroup revealed a significant positive correlation (r = 0.10, 95% CI 0.05 to 0.16, *p* < 0.001).

Additionally, we examined the difference in body fat percentage between EP and NP across 6 studies (n = 573; 269 EP, 304 NP). The pooled analysis showed a significant difference between groups (MD 3.73, 95% CI 0.80 to 6.66, *p* = 0.01), with substantial heterogeneity (*I*^*2*^ = 79%, *p* < 0.001) (Fig. 6c). While a nearly 4% higher body fat percentage could be clinically noteworthy, potentially indicating an increased risk for long-term metabolic issues even when overall BMI appears normal [55, 56], this finding lacked statistical robustness. Specifically, a leave-one-out sensitivity analysis revealed that the overall significance was highly sensitive to individual studies. For instance, excluding Kulecka et al. 2023 [57], DiFrancisco-Donoghue et al. 2025 [58], or Onishchenko et al. 2025 [59] rendered the overall difference non-significant. Notably, this significant difference was not observed within the professional subgroup (MD 2.26, 95% CI –4.11 to 8.63, *p* = 0.49). Consequently, due to the lack of statistical robustness and the observed subgroup variations, the potential clinical implications of these body fat differences must be interpreted with considerable caution. Detailed results of the leave-one-out sensitivity analysis are provided in S1 Table 8.

Regarding other body composition parameters that could not be quantitatively synthesized, several studies reported notable disparities between EP and NP. For instance, DiFrancisco-Donoghue et al. observed significantly lower total lean mass (50.8 ± 4.0 vs 59.8 ± 8.5 kg, *p* = 0.003) in esports participants compared to the comparison group [20]. These findings were corroborated by Kulecka et al., who reported significantly lower muscle mass (27.03 ± 3.98 vs 35.42 ± 4.53 kg, *p* < 0.001) in EP compared to physical education students [57]. However, Giakoni Ramírez et al. found no significant correlation between years of esports experience and body composition parameters (lean body mass, bone mass, and body fat) among professional esports players [23].

#### Musculoskeletal System

**Fig. 7:**
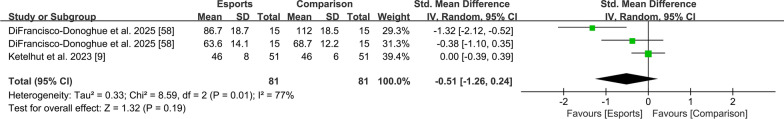
Forest plots of the difference in grip strength between esports players and non-esports players. *CI* Confidence Interval, *df* Degrees of Freedom, *IV* Inverse Variance, *SD* Standard deviation, *Std.* Standardized

Two studies compared grip strength between EP and NP (n = 162; 81 EP, 81 NP). The pooled analysis revealed no significant difference between the groups (SMD –0.51, 95% CI –1.26 to 0.24, *p* = 0.19) (Fig. 7).

Regarding other narratively synthesized parameters, two comparative studies examining young EP versus NP found no significant differences in the prevalence of musculoskeletal pain, including finger, palm, cervical, upper back, and lower back discomfort [21, 35]. However, professional esports players exhibited significantly higher neck and lower back pain rates compared to amateur players. Importantly, gaming posture demonstrated stronger associations with physical complaints than gaming duration [60]. Additionally, semi-professional esports players exhibited significantly reduced thickness of core stabilizing muscles (transversus abdominis, internal oblique, external oblique, and rectus abdominis) [61].

Daily gaming hours exhibited a moderate positive association with functional impairment, as measured by the Boston Carpal Tunnel Questionnaire Functional Severity Scale (r = 0.44, *p* = 0.04) [62]. Consistent with these findings, Uluağaç et al. also demonstrated a significant positive correlation between gaming engagement and upper extremity functional impairments among professional esports athletes [63].

#### Cardiorespiratory Function and Other Physiological Systems

Due to the limited number of studies reporting on these specific physiological parameters, a quantitative synthesis could not be conducted. Thus, these outcomes are synthesized narratively. No significant differences were observed in the self-reported prevalence of visual and auditory symptoms between EP and NP [21]. However, objective visual function assessment revealed significantly impaired parameters in esports athletes, encompassing reduced visual acuity, compromised ocular alignment, and decreased vergence capabilities (*p* < 0.05). These objective visual deficits were associated with a higher prevalence of computer vision syndrome (CVS) in the esports group (*p* < 0.001) [51]. Furthermore, intensive communication during gameplay might pose a risk to vocal health, with approximately 20.7% of esports players experiencing voice problems (e.g., throat dryness and vocal fatigue) associated with prolonged gaming hours and poor environmental air quality [64].

In comparisons of respiratory muscle strength and endurance between EP and age-matched NP, no significant differences were identified [65]. Moreover, no significant between-group differences were detected in systolic blood pressure (t = –0.06; *p* = 0.93; d = –0.01), diastolic blood pressure (t = 0.37; *p* = 0.71; d = 0.07), pulse wave velocity (t = –2.08; *p* = 0.15; d = –0.43), and maximal oxygen consumption (t = –0.11; *p* = 0.92; d = –0.02) [9].

#### Clinical Aspects of Mental Health

**Fig. 8:**
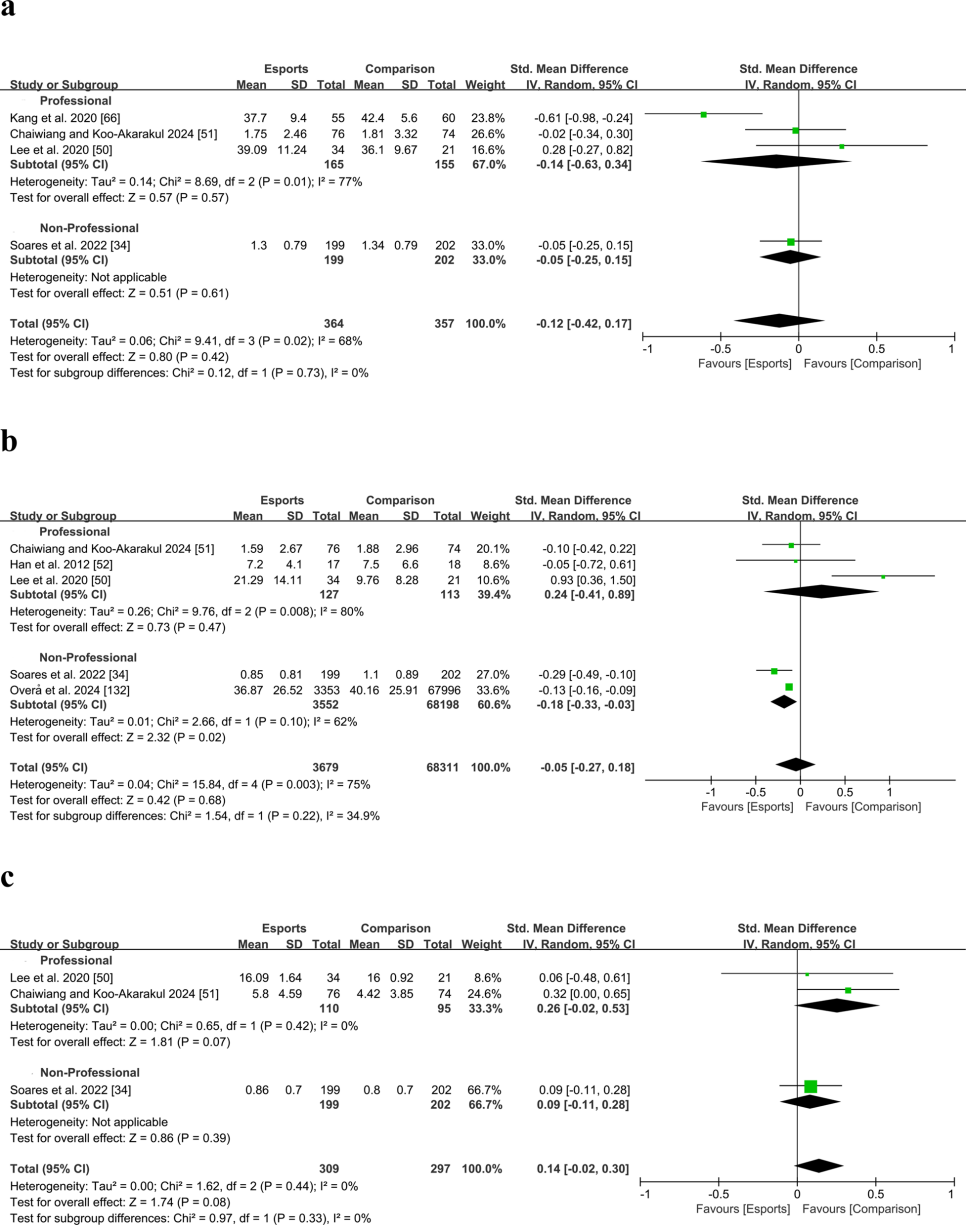
Forest plots of the differences in mental health outcomes between esports players and non-esports players. **a** Anxiety; **b** depression; and **c** stress. *CI* Confidence Interval, *df* Degrees of Freedom, *IV* Inverse Variance, *SD* Standard deviation, *Std.* Standardized

Clinical aspects of mental health in this study were analyzed across four psychological indices: anxiety, depression, stress, and gaming disorder. For anxiety, analysis of four studies (n = 721; 364 EP, 357 NP) showed no significant difference between groups (SMD –0.12, 95% CI –0.42 to 0.17, *p* = 0.42), with substantial heterogeneity (*I*^*2*^ = 68%, *p* = 0.02) (Fig. 8a). Subgroup analyses did not reveal any significant between-group differences. However, upon excluding the study with the greatest heterogeneity (Kang et al. 2020) [66] during the sensitivity analysis, the re-pooled results showed no significant difference (SMD –0.02, 95% CI –0.17 to 0.14, *p* = 0.85), with minimal heterogeneity (*I*^*2*^ = 0%, *p* = 0.54). Although the heterogeneity was substantially reduced, the non-significant nature of the overall effect size remained unchanged. Detailed results of the leave-one-out sensitivity analysis are available in S1 Table 4.

Depression was examined in five studies (n = 71,990; 3,679 EP, 68,311 NP). The pooled analysis revealed no significant difference between groups (SMD –0.05, 95% CI –0.27 to 0.18, *p* = 0.68), with considerable heterogeneity (*I*^*2*^ = 75%, *p* = 0.003) (Fig. 8b). Similarly, subgroup analyses did not show any significant differences between subgroups. However, upon excluding one outlier study [50], the re-pooled results revealed a small but significantly lower level of depressive symptoms among esports participants (SMD –0.13, 95% CI –0.17 to –0.10, *p* < 0.001), with heterogeneity decreasing from 75 to 0%. This raises the possibility that esports participation could be associated with marginally lower depressive symptoms, although the effect size was modest. However, this alteration in significance also indicates a lack of robustness in the current pooled findings, highlighting the need for further research to confirm this result. Detailed results of the leave-one-out sensitivity analysis are provided in S1 Table 5.

Stress levels were analyzed across three studies (n = 606; 309 EP, 297 NP). The analysis indicated no significant difference between groups (SMD 0.14, 95% CI –0.02 to 0.30, *p* = 0.08), with minimal heterogeneity observed (*I*^*2*^ = 0%, *p* = 0.44) (Fig. 8c). Similarly, subgroup analyses did not reveal any significant differences between subgroups.

**Fig. 9:**
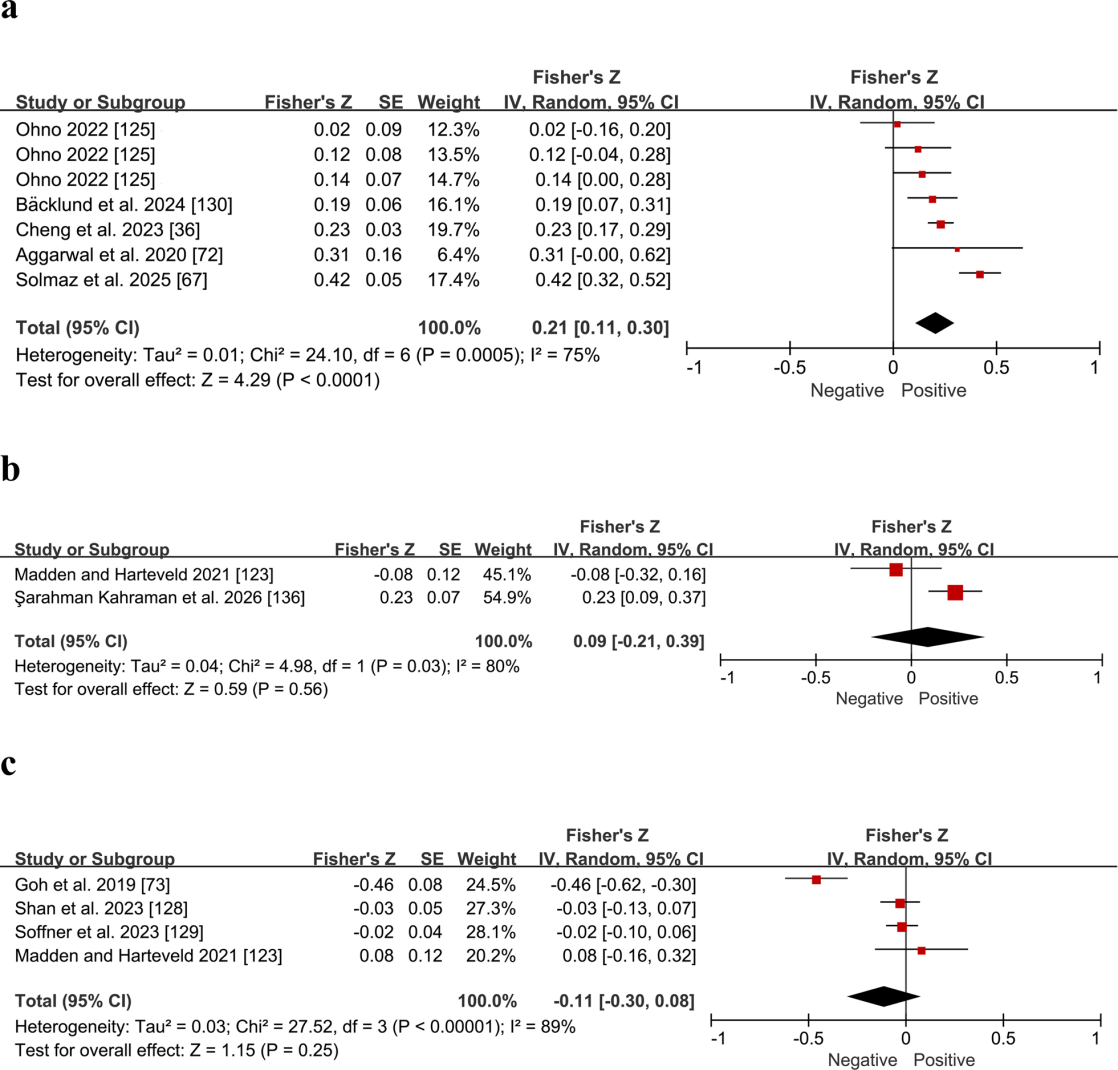
Forest plots of correlations between esports participation and mental health outcomes. **a** Gaming disorder; **b** stress; and **c** psychological well-being. *CI* Confidence Interval, *df* Degrees of Freedom, *IV* Inverse Variance, *SD* Standard deviation, *Std.* Standardized

Five studies were included in the analysis for the correlation between gaming hours and gaming disorder. The pooled effect showed a small but significant positive correlation between gaming hours and gaming disorder (r = 0.10, 95% CI 0.05 to 0.15, *p* < 0.001), with substantial heterogeneity (*I*^*2*^ = 75%, *p* < 0.001) (Fig. 9a). Leave-one-out sensitivity analysis demonstrated that the significance of the pooled effect remained robust regardless of which study was excluded. However, upon excluding the study by Solmaz et al. [67], the re-pooled results maintained significance while heterogeneity was substantially reduced to 30%. Detailed results of the leave-one-out sensitivity analysis are provided in S1 Table 10. Additionally, two studies analyzed the correlation between stress levels and gaming hours. The pooled results did not reveal any significant correlation (r = 0.09, 95% CI –0.21 to 0.37, *p* = 0.56) (Fig. 9b).

Among the studies not included in the quantitative synthesis, Harris et al. similarly found a significant correlation between gaming frequency and gaming disorder (r = 0.36) [68]. Additionally, Mendoza et al. found that expert esports players exhibited significantly higher pre-competition cognitive anxiety (22.44 ± 7.41 vs 15.70 ± 3.99) and cortisol levels (3.75 ± 1.76 vs 2.68 ± 1.48) compared to NP [69]. Luo et al. reported that prolonged esports participation was associated with increased mental fatigue among college students (r = 0.379), with 80.95% of participants experiencing immediate fatigue post-gameplay [70]. Yamamoto et al. demonstrated that nighttime esports training was significantly associated with elevated psychological distress among high-level competitors (OR = 3.80, 95% CI 1.50 to 9.64, *p* = 0.005) [71]. Lastly, by analyzing in-game data, Aggarwal et al. revealed a significant correlation between the number of matches played and adult attention-deficit/hyperactivity disorder (ADHD) symptoms (r = 0.50) [72].

#### Psychological Well-Being

Meta-analysis of four studies revealed no significant overall correlation between esports participation (gaming hours) and psychological well-being (r = –0.11, 95% CI –0.29 to 0.08, *p* = 0.28), with substantial heterogeneity observed across studies (*I*^*2*^ = 89%, *p* < 0.001) (Fig. 9c). Sensitivity analysis demonstrated that although the omission of any single study did not alter the statistical significance of the overall effect, excluding Goh et al. 2019 [73] reduced the heterogeneity to a negligible level (r = –0.02, 95% CI –0.07 to 0.04, *I*^*2*^ = 0). Detailed results of the leave-one-out sensitivity analysis are provided in S1 Table 9.

Furthermore, among the studies not included in the quantitative synthesis, it was found that increased esports engagement did not reduce in-game loneliness but led to heightened post-gaming loneliness among college students [74]. However, EP reported significantly higher levels of life satisfaction [75]. No significant differences were found between esports and non-esports groups in measures of self-regulation and growth mindset [76]. Moreover, analyses revealed that students’ engagement in esports demonstrated significant associations with psychological well-being indicators: a positive relationship emerged with life satisfaction (r = 0.111, *p* < 0.001), while perceived pressure exhibited an inverse correlation (r = –0.143, *p* < 0.001) [36].

#### Social Health

Regarding social connections, comparative analyses yielded mixed findings across studies. While Soares et al. demonstrated significantly lower social connection scores among esports players (M = 4.14 ± 1.15) compared to physical sports players (M = 4.53 ± 1.14), *p* < 0.001 [34], Kelly et al.’s investigation found no significant variations among gaming intensity groups, with one-way ANOVA revealing comparable social connection levels across heavy EP, light EP, and NP for both minors (F = 1.26, *p* = 0.29) and young adults (F = 1.26, *p* = 0.99) [75]. Furthermore, no significant difference in positive youth development was observed [76].

### Publication Bias and Sensitivity Analysis

Detailed results of all sensitivity analyses and funnel plots for publication bias are available in the Supplementary Materials 1 (see S1 Table 3–10 and S1 Fig. 1–15). Visual inspection of the funnel plots did not reveal any obvious asymmetry. However, it is important to note that due to the limited number of included studies for several outcomes (n < 10), some funnel plots lacked sufficient statistical power for definitive qualitative conclusions and should be interpreted with caution. Nevertheless, all plots and analytical results have been provided to ensure full methodological transparency.

## Discussion

This meta-analysis systematically evaluated the associations between esports participation and multidimensional health outcomes across behavioral, physical, mental, and social domains. The review of 53 studies encompassed over 86,000 participants across diverse geographical regions. Our quantitative synthesis of 36 studies revealed no statistically significant between-group differences in most physical health indicators, including BMI, PA levels, sleep duration, and grip strength. Additionally, no significant correlation was observed between gaming hours and sleep quality. Regarding mental health, pooled analyses revealed no significant between-group differences in overall depression, anxiety, or stress parameters. However, sensitivity analysis indicated a potential trend toward lower depression scores among EP when a specific outlier study was excluded. Furthermore, correlational analyses identified no significant association between esports participation and overall psychological well-being or stress, while revealing significant positive correlations with both sedentary behavior and gaming disorder.

Therefore, based on currently available evidence, esports players do not appear to exhibit more health concerns than their non-esports peers, prompting a re-evaluation of conventional negative assumptions regarding esports participation. Our quantitative findings align with recent scoping reviews on esports and health [22], while diverging from earlier research perspectives [3, 18]. This reflects an emerging trend toward a more nuanced and comprehensive understanding of esports participation and players’ health profiles.

Regarding behavioral factors, our analysis revealed comparable PA levels between EP and NP, with no significant differences identified in subgroup analyses between professional and non-professional players. Despite this general parity, some studies suggest that high-level elite esports players may demonstrate greater engagement in physical activity, indicating a potential positive correlation between competitive ranking and PA levels [12, 24]. Although current reports suggest that elite players exercise primarily to maintain overall health [24], emerging evidence shows that physical training has the potential to enhance esports performance, potentially promoting the future integration of PA into daily training routines [4, 77–80]. Additionally, some studies note that highly competitive games closely related to traditional sports themes (particularly sports video games) may increase players’ interest and motivation to engage in PA, suggesting that physical activity levels may also vary among players of different game genres [81–83]. However, current literature still presents inconsistent findings regarding the exact relationship between esports skill levels and physical activity [11, 84]. Therefore, the specific association between esports level and PA warrants further investigation through targeted, high-quality studies in the future. Traditionally, sedentary behavior has often been closely associated with physical inactivity and unhealthy lifestyle habits [85–88]. However, as screen-based activities become a ubiquitous component of modern life, this dramatic lifestyle shift is challenging the traditional paradigm that equates the presence of “sedentary behavior” with “physical inactivity” or poor health habits [89–91]. The complex coexistence of “inevitable sedentary time” and “proactive physical training” exhibited within the esports community may precisely reflect this contemporary lifestyle pattern. Therefore, health assessments of this population require a more comprehensive evaluation of their engagement patterns, intensity, and overall health habits. Additionally, while a recent study suggested that the high PA levels reported by esports players might involve overreporting [11], such discrepancies between subjective and objective measurements are equally common in general population research [27, 28]. Considering the small sample size used for objective validation in that study[11], its external validity is limited. Thus, without direct comparative evidence, one should not over-extrapolate that esports players are uniquely prone to severe reporting biases. Ultimately, this highlights the methodological limitation of heavily relying on self-reported data in current literature, underscoring the urgent need for future studies to incorporate objective measurement tools to obtain accurate PA data and avoid self-reporting bias.

In terms of anthropometric measures, while our meta-analysis found no significant differences in BMI between esports and non-esports participants, a significant positive correlation was observed between esports participation and BMI. This suggests a potential dose–response relationship: although simply engaging in esports does not inherently lead to a higher BMI compared to the general population, the intensity and duration of play are positively linked to BMI increases. Such nuances are often obscured in broad binary comparisons (players vs. non-players) due to the widely varying levels of engagement within the esports group. Additionally, preliminary results suggest that the body fat of esports players might be higher than that of non-players (especially in non-professional comparisons). However, substantial heterogeneity was observed in studies examining body fat parameters, and the sensitivity analysis showed these findings to be highly non-robust. Thus, larger-scale, higher-quality data are required to validate these comparisons. Furthermore, most existing studies failed to adequately control for potential confounding variables, such as sex distribution, gaming patterns, and other behavioral habits. Given the well-documented sex differences in body composition parameters, varying male-to-female ratios across studies may introduce systematic bias into the reported anthropometric measurements.

As in traditional professional sports and conventional sedentary occupations, musculoskeletal disorders have been documented among professional esports athletes [92, 93]. However, when exploring broader esports populations, the current literature lacks stratification by practice level, competitive ranking, or professional status [21, 22]. This methodological limitation hampers our understanding of how musculoskeletal symptoms vary across different participation levels. Therefore, future studies must adopt standardized reporting models for players’ professional status and competitive levels to accurately stratify these health risks [29, 94]. Notably, emerging evidence suggests that posture plays a more critical role in the development of musculoskeletal symptoms, highlighting the paramount importance of ergonomic considerations in esports health outcomes [21, 60, 89]. Given the significant correlation between posture and musculoskeletal symptoms, future research must precisely differentiate the health impacts of esports participation itself from the postural factors associated with it. This necessitates rigorous control and quantification of postural habits in research designs, alongside considerations of professional status and engagement patterns. By isolating these variables, researchers can clearly determine whether the esports activity itself or its accompanying postural habits play a more decisive role in musculoskeletal health. Furthermore, beyond general musculoskeletal issues, specific localized conditions, particularly carpal tunnel syndrome, De Quervain’s tenosynovitis, and computer vision syndrome (CVS), warrant special attention [51, 62, 95]. A few studies have linked these symptoms to prolonged or intensive esports participation, indicating a future need for targeted preventive measures specifically addressing these conditions.

While our analysis identified no significant differences in overall sleep duration or insomnia prevalence between groups, existing literature reveals a consistent pattern of prolonged sleep latency among EP [96, 97]. Additionally, observational data indicate that each additional hour of weekly esports engagement correlates with an increased risk of night eating syndrome [53]. These sleep–wake rhythm alterations likely stem from the esports ecosystem, which includes evening competition schedules, prolonged training sessions, and a highly active nocturnal gaming culture across different time zones [96]. Live streaming, an integral component of esports careers, may further exacerbate this pattern, as streams are typically scheduled during evening hours to maximize audience reach and interaction [98, 99]. Moreover, sleep patterns appear to vary based on game modality, team requirements, and specific game characteristics [96]. Consequently, esports participation modes and the degree of professionalization may be differentially associated with these specific health outcomes.

Regarding psychological and social health outcomes, although previous research linked esports participation to common mental health conditions (e.g., stress, anxiety, and depression), our meta-analysis observed no statistically significant differences in these parameters between multi-level EP and NP. However, given the potential heterogeneity across included studies, this lack of significance should be interpreted with caution. Individual characteristics (e.g., coping mechanisms and mental toughness) and environmental factors (e.g., media, team, and competition settings) may moderate these relationships [22, 44, 100–102]. Furthermore, the potentially lower depression scores observed among recreational esports participants might be attributed to previous findings that esports gaming serves adaptive functions in stress management and emotion regulation [103–105]. Given the current lack of robustness, confirming these potential protective effects requires more inclusive and longitudinal research. Conversely, consistent with prior studies [106–108], we also identified potential gaming disorder risks associated with excessive gaming. Since current results are primarily based on cross-sectional data, the causal direction between gaming duration and gaming disorder requires longitudinal verification. The possibility of reverse causality cannot be ruled out, namely, that an individual’s predisposition to gaming disorder drives longer gaming hours, rather than gaming hours solely causing gaming disorder. Studies also indicate that psychological traits like aggression, self-control, and narcissism may predispose certain individuals to online gaming addiction, with distinct patterns observed between professional and recreational players [109, 110]. These results underscore the importance of distinguishing participation modes and accounting for psychological traits in future mental health research, thereby providing a more nuanced and actionable basis for healthy esports guidelines.

Overall, current evidence regarding the associations between esports and health outcomes lacks generalizability and certainty, exhibiting high heterogeneity. First, this may partly stem from definitional ambiguities (e.g., whether “esports players” refers exclusively to professionals). Professionals face unique conditions, intensive training, sustained competitive pressure, and specific occupational stressors, rarely encountered by recreational players [111, 112]. Extrapolating the health profiles of professionals to the broader casual esports community requires extreme caution, analogous to how injury risks in traditional professional sports represent occupation-specific hazards rather than activity-inherent dangers [113, 114]. Second, there is a lack of detailed reporting on gaming behaviors, including specific genres, purposes, and forms of play. With technological advancements, emerging forms like virtual sports have been integrated into mainstream esports. These variants differ significantly from traditional esports, involving distinct physical engagement patterns, cognitive demands, and physiological responses [29, 115–117]. Furthermore, previous studies show that sedentary behaviors with different forms or purposes yield varying health impacts and cannot be treated as a uniform risk factor [89]. For instance, occupational sitting and television viewing exhibit different health associations, likely due to confounders like snacking, socioeconomic status, and patterns of interrupting sedentary time [118–120]. When assessing sedentary behavior, its specific context and accompanying factors must be considered, rather than solely focusing on duration. This highlights the importance of cautiously interpreting how various game-related factors moderate health outcomes. Ultimately, these methodological limitations, coupled with the complex coexistence of “inevitable sedentary time” and “proactive physical training”, jointly contribute to the significant heterogeneity in current evidence [21, 22, 29]. Consequently, existing evidence remains insufficient to establish a definitive link between esports participation and health deterioration or negative lifestyle changes.

In summary, these findings indicate that the relationship between esports participation and health outcomes is far more complex than previously assumed, necessitating comprehensive research methodologies that account for participation intensity, gaming modalities, and individual characteristics. Given that the intensity, duration, and frequency of engagement likely moderate these health associations, developing stratified, evidence-based practical guidelines is imperative. For general participants, health guidelines regarding play duration, intensity, and frequency should be established to promote sustainable engagement. Conversely, for professional athletes, highly targeted training and preventive regimens must be developed, training models must be scientifically optimized, and close attention must be paid to their mental health. This will ensure their physical well-being and career longevity amidst competitive excellence. To achieve this, first, future research should prioritize establishing standardized definitions and rigorous measurement protocols, implementing strict controls for confounding variables, and closely examining potential moderators. This approach will provide more objective and targeted empirical evidence, thereby enhancing the overall quality and systematicity of esports research. Second, to overcome the limitations of current cross-sectional data, future studies must prioritize longitudinal investigations. This will enable researchers to better understand the dose–response and causal relationships between esports participation and health outcomes across different populations, providing a robust scientific foundation for evidence-based interventions that optimize player health while fostering the sustainable development of the esports industry.

## Strengths and Limitations

This systematic review and meta-analysis adhered to rigorous methodological standards, following PRISMA and JBI frameworks to ensure a systematic and transparent research process. A key strength of our study lies in its stringent screening criteria specifically tailored for esports, carefully distinguishing it from general video gaming. This precise differentiation addresses a significant gap in the current literature where the boundaries between esports and general video gaming often remain ambiguous or inadequately delineated. Additionally, we conducted subgroup analyses comparing professional esports athletes with non-professional players, acknowledging the distinct behavioral patterns and health implications associated with the intensive training regimens and high-stakes competitive environments characteristic of professional esports. Our study presents the first systematic quantitative synthesis examining the relationship between esports participation and comprehensive health outcomes. In contrast to previous scoping reviews that provided narrative summaries, our meta-analytic approach, incorporating a substantial pooled sample size, offers statistical integration of effect sizes for more precise outcome estimates. This rigorous methodology not only provides the most comprehensive evaluation to date but also establishes a foundation for future research in this emerging field.

However, several limitations of our study should be acknowledged. First, for certain health outcomes, such as sleep quality and grip strength, the limited number of eligible studies may have affected the precision and reliability of our effect size estimates. This paucity precluded further subgroup analyses to identify potential moderating variables and formal assessments of publication bias.

Second, the pooled results for specific indicators, such as body fat percentage and gaming disorder, demonstrated substantial heterogeneity. This reduces the overall certainty of the synthesized findings, suggesting that the integrated results of current evidence should be interpreted with caution. A qualitative review of the included studies suggests that this observed heterogeneity likely stems primarily from the diversity of study characteristics, including variations in inclusion/exclusion criteria for esports players, sample sizes, participants’ gaming levels, patterns, and individual differences. However, it must be noted that this is merely a descriptive observation rather than a definitive quantitative conclusion. Future meta-analyses, supported by a larger body of eligible studies, are required to conduct reliable subgroup analyses and meta-regressions to definitively pinpoint the exact sources of heterogeneity.

Third, inconsistent reporting of essential variables across primary studies posed a significant challenge: many studies failed to specify participants’ game genres, gaming intensity, professional status, or even demographic characteristics. Consequently, we were unable to conduct subgroup analyses to explore genre-specific or demographic-specific associations with health outcomes. Additionally, this lack of detailed information limited our ability to further verify whether the participants in some primary studies strictly met the criteria for esports players. With the recent emergence of more detailed and standardized definitions regarding the competitive and professional status of esports [94], we recommend that future studies adopt these standardized guidelines when reporting players’ professional status and skill levels. This will provide a crucial foundation for the standardization of esports research and the specificity of future findings. Notably, the predominant representation of male participants in most included studies limits the generalizability of our findings to female esports players.

Fourth, the current findings are primarily based on cross-sectional data, which only allows for an exploratory analysis of the associations between esports participation and health outcomes. Therefore, definitive causal inferences cannot be drawn. Future longitudinal studies are warranted to further elucidate these causal relationships.

Finally, the vast majority of current studies rely on subjective recall to assess players' esports behaviors, including participation level, years of experience, and gaming patterns. Similarly, health outcomes such as musculoskeletal issues and mental health conditions are typically evaluated using self-reported questionnaires, which may introduce recall bias. Future research should employ more objective and precise measurement tools for accurate assessments.

As more high-quality studies are published in this field, future comprehensive meta-analyses may provide more robust evidence, enable proper assessment of publication bias, and explore potential moderating variables. Future empirical research examining the associations between esports and health outcomes should enhance methodological rigor by systematically documenting and controlling for potential moderators, thus allowing for more precise effect size estimates and a deeper understanding of these associations.

## Conclusion

This systematic review and meta-analysis present a comprehensive evidence synthesis regarding the relationship between esports participation and health outcomes. While current evidence suggests that esports players may exhibit health profiles largely comparable to their non-esports peers across multiple indicators, these findings must be interpreted with caution due to high heterogeneity and significant evidence gaps in the existing literature. Notably, while increased engagement intensity appears to be associated with higher risks of gaming disorder and sedentary behavior, differentiated patterns emerged between professional and recreational participants.

These findings indicate that the relationship between esports participation and health is highly complex. Future research must prioritize establishing standardized operational definitions for the inclusion and exclusion of esports players, as well as unified classification and reporting standards. Furthermore, longitudinal investigations are urgently needed to elucidate the dose–response and causal relationships between esports participation and health outcomes.

In practice, there is a critical need for the esports industry to establish standardized health monitoring protocols and promote transparent data sharing. Esports organizations, team managers, and healthcare professionals should collaborate to develop stratified, evidence-based, practical guidelines. For general participants, guidelines regarding play duration and frequency should promote sustainable and healthy engagement. Conversely, for professional athletes, highly targeted training and preventive regimens must be developed, and close attention must be paid to their mental health. Implementing these targeted prevention and intervention strategies will be crucial in safeguarding players’ long-term physical and mental well-being while fostering the sustainable development of the esports industry.

## Supplementary Information

Additional file 1.

## Data Availability

The datasets analyzed in this review are available from the corresponding author upon reasonable request.

## Abbreviations

ACG: Action video game
ADHD: Attention-deficit/hyperactivity disorder
BMI: Body mass index
CI: Confidence intervals
CVS: Computer vision syndrome
EP: Esports participants
FG: Fighting games
FPS: First-person shooter
JBI: Joanna Briggs Institute
MD: Mean difference
MOBA: Multiplayer online battle arena
NG: Non-Video gamers
NP: Non-Esports participants
NR: Not reported
PA: Physical activity
Pro: Professional
PROSPERO: International Prospective Register of Systematic Reviews
PRISMA: Preferred Reporting Items for Systematic Reviews and Meta-analyses
RTS: Real-time strategy games
SCG: Strategy card games
SMD: Standardized mean difference
SVG: Sports video games
