# Supplementary material for: Association Between Esports Participation and Health: A Systematic Review and Meta-analysis
Source: Sports Med Open. 2026 Jul 29;12:108. doi: 10.1186/s40798-026-01083-0 (PMC13421603; doi:10.1186/s40798-026-01083-0)
Supplement: Supplementary file 1 — Additional file 1. [file 40798_2026_1083_MOESM1_ESM.docx]

|  |  | **Page** |
| --- | --- | --- |
| **Supplementary Information 1** | PRISMA checklist | 2-5 |
| **Supplementary Information 2** | Search strategy | 6-8 |
| **Supplementary Table 1** | Quality assessment of included studies | 9-10 |
| **Supplementary Table 2** | Leave-one-out sensitivity analysis of body mass index comparison | 11 |
| **Supplementary Table 3** | Leave-one-out sensitivity analysis of physical activity comparison | 12 |
| **Supplementary Table 4** | Leave-one-out sensitivity analysis of anxiety comparison | 13 |
| **Supplementary Table 5** | Leave-one-out sensitivity analysis of depression comparison | 14 |
| **Supplementary Table 6** | Leave-one-out sensitivity analysis of stress comparison | 15 |
| **Supplementary Table 7** | Leave-one-out sensitivity analysis of sleep duration comparison | 16 |
| **Supplementary Table 8** | Leave-one-out sensitivity analysis of fat percentage comparison | 17 |
| **Supplementary Table 9** | Leave-one-out sensitivity analysis of correlation between participation and psychological well-being | 18 |
| **Supplementary Table 10** | Leave-one-out sensitivity analysis of correlation between participation and gaming disorder | 19 |
| **Supplementary Figure 1** | Funnel plot for body mass index comparison | 20 |
| **Supplementary Figure 2** | Funnel plot for physical activity comparison | 21 |
| **Supplementary Figure 3** | Funnel plot for depression comparison | 22 |
| **Supplementary Figure 4** | Funnel plot for anxiety comparison | 23 |
| **Supplementary Figure 5** | Funnel plot for stress comparison | 24 |
| **Supplementary Figure 6** | Funnel plot for sleep duration comparison | 25 |
| **Supplementary Figure 7** | Funnel plot for fat percentage comparison | 26 |
| **Supplementary Figure 8** | Funnel plot for grip strength comparison | 27 |
| **Supplementary Figure 9** | Funnel plot for the correlation between participation and psychological well-being | 28 |
| **Supplementary Figure 10** | Funnel plot for the correlation between participation and gaming disorder | 29 |
| **Supplementary Figure 11** | Funnel plot for the correlation between participation and body mass index | 30 |
| **Supplementary Figure 12** | Funnel plot for the correlation between participation and sedentary behavior | 31 |
| **Supplementary Figure 13** | Funnel plot for the correlation between participation and physical activity | 32 |
| **Supplementary Figure 14** | Funnel plot for the correlation between participation and stress | 33 |
| **Supplementary Figure 15** | Funnel plot for the correlation between participation and sleep quality | 34 |

**Supplemental Material 1**

| **Section and Topic** | **Item #** | **Checklist item** | **Location where item is reported** |
| --- | --- | --- | --- |
| **TITLE** | | |  |
| Title | 1 | Identify the report as a systematic review. | 1 |
| **ABSTRACT** | | |  |
| Abstract | 2 | See the PRISMA 2020 for Abstracts checklist. | 3 |
| **INTRODUCTION** | | |  |
| Rationale | 3 | Describe the rationale for the review in the context of existing knowledge. | 4-6 |
| Objectives | 4 | Provide an explicit statement of the objective(s) or question(s) the review addresses. | 6 |
| **METHODS** | | |  |
| Eligibility criteria | 5 | Specify the inclusion and exclusion criteria for the review and how studies were grouped for the syntheses. | 7-8 |
| Information sources | 6 | Specify all databases, registers, websites, organisations, reference lists and other sources searched or consulted to identify studies. Specify the date when each source was last searched or consulted. | 6 |
| Search strategy | 7 | Present the full search strategies for all databases, registers and websites, including any filters and limits used. | 6-7 + Supplemental Information 1 |
| Selection process | 8 | Specify the methods used to decide whether a study met the inclusion criteria of the review, including how many reviewers screened each record and each report retrieved, whether they worked independently, and if applicable, details of automation tools used in the process. | 7 |
| Data collection process | 9 | Specify the methods used to collect data from reports, including how many reviewers collected data from each report, whether they worked independently, any processes for obtaining or confirming data from study investigators, and if applicable, details of automation tools used in the process. | 8 |
| Data items | 10a | List and define all outcomes for which data were sought. Specify whether all results that were compatible with each outcome domain in each study were sought (e.g. for all measures, time points, analyses), and if not, the methods used to decide which results to collect. | 6-8 |
|  | 10b | List and define all other variables for which data were sought (e.g. participant and intervention characteristics, funding sources). Describe any assumptions made about any missing or unclear information. | 6-8 |
| Study risk of bias assessment | 11 | Specify the methods used to assess risk of bias in the included studies, including details of the tool(s) used, how many reviewers assessed each study and whether they worked independently, and if applicable, details of automation tools used in the process. | 8 |
| Effect measures | 12 | Specify for each outcome the effect measure(s) (e.g. risk ratio, mean difference) used in the synthesis or presentation of results. | 8-9 |
| Synthesis methods | 13a | Describe the processes used to decide which studies were eligible for each synthesis (e.g. tabulating the study intervention characteristics and comparing against the planned groups for each synthesis (item #5)). | 8-9 |
|  | 13b | Describe any methods required to prepare the data for presentation or synthesis, such as handling of missing summary statistics, or data conversions. | 8-9 |
|  | 13c | Describe any methods used to tabulate or visually display results of individual studies and syntheses. | 8-9 |
|  | 13d | Describe any methods used to synthesize results and provide a rationale for the choice(s). If meta-analysis was performed, describe the model(s), method(s) to identify the presence and extent of statistical heterogeneity, and software package(s) used. | 8-9 |
|  | 13e | Describe any methods used to explore possible causes of heterogeneity among study results (e.g. subgroup analysis, meta-regression). | 8-9 |
|  | 13f | Describe any sensitivity analyses conducted to assess robustness of the synthesized results. | 8-9 |
| Reporting bias assessment | 14 | Describe any methods used to assess risk of bias due to missing results in a synthesis (arising from reporting biases). | 8-9 |
| Certainty assessment | 15 | Describe any methods used to assess certainty (or confidence) in the body of evidence for an outcome. | na |
| **RESULTS** | | |  |
| Study selection | 16a | Describe the results of the search and selection process, from the number of records identified in the search to the number of studies included in the review, ideally using a flow diagram. | 9-10 |
|  | 16b | Cite studies that might appear to meet the inclusion criteria, but which were excluded, and explain why they were excluded. | Table 1, Figure 2 |
| Study characteristics | 17 | Cite each included study and present its characteristics. | Table 1, Figure 2 |
| Risk of bias in studies | 18 | Present assessments of risk of bias for each included study. | 10-11 |
| Results of individual studies | 19 | For all outcomes, present, for each study: (a) summary statistics for each group (where appropriate) and (b) an effect estimate and its precision (e.g. confidence/credible interval), ideally using structured tables or plots. | 10, Table 1, Figure 3-9 |
| Results of syntheses | 20a | For each synthesis, briefly summarise the characteristics and risk of bias among contributing studies. | 7-13 |
|  | 20b | Present results of all statistical syntheses conducted. If meta-analysis was done, present for each the summary estimate and its precision (e.g. confidence/credible interval) and measures of statistical heterogeneity. If comparing groups, describe the direction of the effect. | 11-16 |
|  | 20c | Present results of all investigations of possible causes of heterogeneity among study results. | 11-16 |
|  | 20d | Present results of all sensitivity analyses conducted to assess the robustness of the synthesized results. | 11-16 |
| Reporting biases | 21 | Present assessments of risk of bias due to missing results (arising from reporting biases) for each synthesis assessed. | 16 |
| Certainty of evidence | 22 | Present assessments of certainty (or confidence) in the body of evidence for each outcome assessed. | na |
| **DISCUSSION** | | |  |
| Discussion | 23a | Provide a general interpretation of the results in the context of other evidence. | 16-17 |
|  | 23b | Discuss any limitations of the evidence included in the review. | 17-20 |
|  | 23c | Discuss any limitations of the review processes used. | 17-20 |
|  | 23d | Discuss implications of the results for practice, policy, and future research. | 17-20 |
| **OTHER INFORMATION** | | |  |
| Registration and protocol | 24a | Provide registration information for the review, including register name and registration number, or state that the review was not registered. | 3 |
|  | 24b | Indicate where the review protocol can be accessed, or state that a protocol was not prepared. | na |
|  | 24c | Describe and explain any amendments to information provided at registration or in the protocol. | 3 |
| Support | 25 | Describe sources of financial or non-financial support for the review, and the role of the funders or sponsors in the review. | 24 |
| Competing interests | 26 | Declare any competing interests of review authors. | 24 |
| Availability of data, code and other materials | 27 | Report which of the following are publicly available and where they can be found: template data collection forms; data extracted from included studies; data used for all analyses; analytic code; any other materials used in the review. | 24 |

*From:*  Page MJ, McKenzie JE, Bossuyt PM, Boutron I, Hoffmann TC, Mulrow CD, et al. The PRISMA 2020 statement: an updated guideline for reporting systematic reviews. BMJ 2021;372:n71. doi: 10.1136/bmj.n71. This work is licensed under CC BY 4.0. To view a copy of this license, visit <https://creativecommons.org/licenses/by/4.0/>

**Search Strategy**

**Databases:**

PubMed, Web of Science, Scopus, SPORTDiscus, MEDLINE, ProQuest, Esports Research Network.

**Search period:**

Inception to May 13, 2026.

**Search terms:**

(esport* OR “virtual sport*” OR “cyber sport*” OR “competitive video gam*” OR “competitive gam*” OR “electronic sport*” OR “e-sport*” OR “professional gam*” OR “professional video gam*” OR “pro gam*” OR “multiplayer online battle arena” OR “first person shooter” OR “shooting gam*” OR “sports video gam*” OR “strategy card gam*” OR “real-time strategy” OR “fighting gam*” OR “Battle Royale” OR “League of Legends” OR “Counter-Strike” OR “Valorant” OR “Overwatch” OR “StarCraft” OR “Rocket League” OR “Hornor of Kings” OR “Mobile Legends”) AND (health OR “body mass” OR illness OR injur* OR pain* OR fitness OR disorder OR mortality OR morbidity OR lifestyle OR "well-being" OR wellbeing OR anxiety OR stress OR depression OR loneliness OR “physical activity” OR sedentar* OR nutrition OR eating OR sleep OR fatigue OR exercise OR “physical exertion” OR conditioning OR “physical training” OR physiology) NOT (esporte* OR esporti* OR gamma*).

**Detailed search strings in each database:**

| **Database** | **Search** | **Results** |
| --- | --- | --- |
| PubMed | ((("esport*"[Title/Abstract] OR "virtual sport*"[Title/Abstract] OR "cyber sport*"[Title/Abstract] OR "competitive video gam*"[Title/Abstract] OR "competitive gam*"[Title/Abstract] OR "electronic sport*"[Title/Abstract] OR "e sport*"[Title/Abstract] OR "professional gam*"[Title/Abstract] OR "professional video gam*"[Title/Abstract] OR "pro gam*"[Title/Abstract] OR "multiplayer online battle arena"[Title/Abstract] OR "first person shooter"[Title/Abstract] OR "shooting gam*"[Title/Abstract] OR "sports video gam*"[Title/Abstract] OR "real-time strategy"[Title/Abstract] OR "fighting gam*"[Title/Abstract] OR "Battle Royale"[Title/Abstract] OR "League of Legends"[Title/Abstract] OR "Counter-Strike"[Title/Abstract] OR "Valorant"[Title/Abstract] OR "Overwatch"[Title/Abstract] OR "StarCraft"[Title/Abstract] OR "Honor of Kings"[Title/Abstract]) AND ("health"[Title/Abstract] OR "body mass"[Title/Abstract] OR "illness"[Title/Abstract] OR "injur*"[Title/Abstract] OR "pain*"[Title/Abstract] OR "fitness"[Title/Abstract] OR "disorder"[Title/Abstract] OR "mortality"[Title/Abstract] OR "morbidity"[Title/Abstract] OR "lifestyle"[Title/Abstract] OR "well-being"[Title/Abstract] OR "wellbeing"[Title/Abstract] OR "anxiety"[Title/Abstract] OR "stress"[Title/Abstract] OR "depression"[Title/Abstract] OR "loneliness"[Title/Abstract] OR "physical activity"[Title/Abstract] OR "sedentar*"[Title/Abstract] OR "nutrition"[Title/Abstract] OR "eating"[Title/Abstract] OR "sleep"[Title/Abstract] OR "fatigue"[Title/Abstract] OR "exercise"[Title/Abstract] OR "physical exertion"[Title/Abstract] OR "conditioning"[Title/Abstract] OR "physical training"[Title/Abstract] OR "physiology"[Title/Abstract])) NOT ("esporte*"[Title/Abstract] OR "esporti*"[Title/Abstract] OR "gamma*"[Title/Abstract])) AND (chinese[Filter] OR english[Filter]) | 607 |
| Web of Science | (TS=(esport* OR “virtual sport*” OR “cyber sport*” OR “competitive video gam*” OR “competitive gam*” OR “electronic sport*” OR “e-sport*” OR “professional gam*” OR “professional video gam*” OR “pro gam*” OR “multiplayer online battle arena” OR “first person shooter” OR “shooting gam*” OR “sports video gam*” OR “strategy card gam*” OR “real-time strategy” OR “fighting gam*” OR “Battle Royale” OR “League of Legends” OR “Counter-Strike” OR “Valorant” OR “Overwatch” OR “StarCraft” OR “Rocket League” OR “Honor of Kings” OR “Mobile Legends”) AND TS=(health OR “body mass” OR illness OR injur* OR pain* OR fitness OR disorder OR mortality OR morbidity OR lifestyle OR "well-being" OR wellbeing OR anxiety OR stress OR depression OR loneliness OR “physical activity” OR sedentar* OR nutrition OR eating OR sleep OR fatigue OR exercise OR “physical exertion” OR conditioning OR “physical training” OR physiology) NOT TS=(esporte* OR esporti* OR gamma*)) AND ((LA==("ENGLISH" OR "CHINESE")) NOT (DT==("EDITORIAL MATERIAL" OR "MEETING ABSTRACT" OR "LETTER" OR "CORRECTION" OR "REVIEW" OR "PROCEEDINGS PAPER"))) | 1368 |
| MEDLINE | esport* OR “virtual sport*” OR “cyber sport*” OR “competitive video gam*” OR “competitive gam*” OR “electronic sport*” OR “e-sport*” OR “professional gam*” OR “professional video gam*” OR “pro gam*” OR “multiplayer online battle arena” OR “first person shooter” OR “shooting gam*” OR “sports video gam*” OR “strategy card gam*” OR “real-time strategy” OR “fighting gam*” OR “Battle Royale” OR “League of Legends” OR “Counter-Strike” OR “Valorant” OR “Overwatch” OR “StarCraft” OR “Rocket League” OR “Honor of Kings” OR “Mobile Legends” (Topic) and health OR “body mass” OR illness OR injur* OR pain* OR fitness OR disorder OR mortality OR morbidity OR lifestyle OR "well-being" OR wellbeing OR anxiety OR stress OR depression OR loneliness OR “physical activity” OR sedentar* OR nutrition OR eating OR sleep OR fatigue OR exercise OR “physical exertion” OR conditioning OR “physical training” OR physiology (Topic) not esporte* OR esporti* OR gamma* (Topic) and English or Chinese (Languages) and Systematic Review or Review Article or Letter or Comment or Meta Analysis or Editorial or Case Reports or Scoping Review or Dataset or Validation Study or Congress or English Abstract or Consensus Development Conference (Exclude – Publication Type) | 751 |
| Scopus | ( TITLE-ABS-KEY ( esport* OR "virtual sport*" OR "cyber sport*" OR "competitive video gam*" OR "competitive gam*" OR "electronic sport*" OR "e-sport*" OR "professional gam*" OR "professional video gam*" OR "pro gam*" OR "multiplayer online battle arena" OR "first person shooter" OR "shooting gam*" OR "sports video gam*" OR "strategy card gam*" OR "real-time strategy" OR "fighting gam*" OR "Battle Royale" OR "League of Legends" OR "Counter-Strike" OR "Valorant" OR "Overwatch" OR "StarCraft" OR "Rocket League" OR "Honor of Kings" OR "Mobile Legends" ) AND TITLE-ABS-KEY ( health OR "body mass" OR illness OR injur* OR pain* OR fitness OR disorder OR mortality OR morbidity OR lifestyle OR "well-being" OR wellbeing OR anxiety OR stress OR depression OR loneliness OR "physical activity" OR sedentar* OR nutrition OR eating OR sleep OR fatigue OR exercise OR "physical exertion" OR conditioning OR "physical training" OR physiology ) AND NOT TITLE-ABS-KEY ( esporte* OR esporti* OR gamma* ) ) AND ( LIMIT-TO ( LANGUAGE , "English" ) ) AND ( EXCLUDE ( DOCTYPE , "cp" ) OR EXCLUDE ( DOCTYPE , "re" ) OR EXCLUDE ( DOCTYPE , "cr" ) OR EXCLUDE ( DOCTYPE , "le" ) OR EXCLUDE ( DOCTYPE , "no" ) OR EXCLUDE ( DOCTYPE , "ed" ) OR EXCLUDE ( DOCTYPE , "er" ) OR EXCLUDE ( DOCTYPE , "dp" ) ) | 1242 |
| SportDiscus | XB (esport* OR “virtual sport*” OR “cyber sport*” OR “competitive video gam*” OR “competitive gam*” OR “electronic sport*” OR “e-sport*” OR “professional gam*” OR “professional video gam*” OR “pro gam*” OR “multiplayer online battle arena” OR “first person shooter” OR “shooting gam*” OR “sports video gam*” OR “strategy card gam*” OR “real-time strategy” OR “fighting gam*” OR “Battle Royale” OR “League of Legends” OR “Counter-Strike” OR “Valorant” OR “Overwatch” OR “StarCraft” OR “Rocket League” OR “Honor of Kings” OR “Mobile Legends”) AND XB (health OR “body mass” OR illness OR injur* OR pain* OR fitness OR disorder OR mortality OR morbidity OR lifestyle OR "well-being" OR wellbeing OR anxiety OR stress OR depression OR loneliness OR “physical activity” OR sedentar* OR nutrition OR eating OR sleep OR fatigue OR exercise OR “physical exertion” OR conditioning OR “physical training” OR physiology) NOT XB (esporte* OR esporti* OR gamma*) | 256 |
| ProQuest | **S1:** abstract(esport* OR “virtual sport*” OR “cyber sport*” OR “competitive video gam*” OR “competitive gam*” OR “electronic sport*” OR “e-sport*” OR “professional gam*” OR “professional video gam*” OR “pro gam*” OR “multiplayer online battle arena” OR “first person shooter” OR “shooting gam*” OR “sports video gam*” OR “strategy card gam*” OR “real-time strategy” OR “fighting gam*” OR “Battle Royale” OR “League of Legends” OR “Counter-Strike” OR “Valorant” OR “Overwatch” OR “StarCraft” OR “Rocket League” OR “Honor of Kings” OR “Mobile Legends”) AND abstract(health OR “body mass” OR illness OR injur* OR pain* OR fitness OR disorder OR mortality OR morbidity OR lifestyle OR "well-being" OR wellbeing OR anxiety OR stress OR depression OR loneliness OR “physical activity” OR sedentar* OR nutrition OR eating OR sleep OR fatigue OR exercise OR “physical exertion” OR conditioning OR “physical training” OR physiology) AND abstract(esporte* OR esporti* OR gamma*);  **S2:** title(esport* OR “virtual sport*” OR “cyber sport*” OR “competitive video gam*” OR “competitive gam*” OR “electronic sport*” OR “e-sport*” OR “professional gam*” OR “professional video gam*” OR “pro gam*” OR “multiplayer online battle arena” OR “first person shooter” OR “shooting gam*” OR “sports video gam*” OR “strategy card gam*” OR “real-time strategy” OR “fighting gam*” OR “Battle Royale” OR “League of Legends” OR “Counter-Strike” OR “Valorant” OR “Overwatch” OR “StarCraft” OR “Rocket League” OR “Honor of Kings” OR “Mobile Legends”) AND title(health OR “body mass” OR illness OR injur* OR pain* OR fitness OR disorder OR mortality OR morbidity OR lifestyle OR "well-being" OR wellbeing OR anxiety OR stress OR depression OR loneliness OR “physical activity” OR sedentar* OR nutrition OR eating OR sleep OR fatigue OR exercise OR “physical exertion” OR conditioning OR “physical training” OR physiology) AND title(esporte* OR esporti* OR gamma*);  **S1 OR S2** (Language (Inclusion):English OR Chinese; Document Type (Exclusion): News, Conference proceedings, Reports, Evidence-based healthcare, Blogs, Commentaries, General information, Reviews, Editorials, Literature reviews, Case studies, Fiction, Instructional materials/Guidelines, Interviews, Speeches/Lectures, Cover stories, Industry reports, Undetermined.). | 799 |

**Note:** Due to the technical limitations of the Esports Research Network database, which does not support advanced search functionality, we conducted a simplified search strategy using only health-related keywords. Relevant articles were then manually identified and screened from these search results. This manual approach, while more labor-intensive, ensured comprehensive coverage of pertinent literature within this specialized database.

|  |  |
| --- | --- |

**Supplementary Table 1 Quality assessment of included studies (Joanna Briggs Quality Assessment)**

| **Study** | **1** | **2** | **3** | **4** | **5** | **6** | **7** | **8** | **Total (Y%)** |
| --- | --- | --- | --- | --- | --- | --- | --- | --- | --- |
| Aggarwal et al. 2020 | U | Y | Y | Y | N | N | Y | U | 50 |
| Bäcklund et al. 2024 | Y | Y | N | Y | Y | Y | Y | Y | 87.5 |
| Bayrakdar et al. 2020 | U | Y | Y | N | N | N | Y | Y | 50 |
| Chaiwiang and Koo-Akarakul 2024 | Y | U | Y | Y | N | N | Y | Y | 62.5 |
| Cheng et al. 2023 | U | Y | N | Y | Y | Y | Y | Y | 75 |
| Cueva-Reguera et al. 2023 | Y | U | N | N | N | N | Y | Y | 37.5 |
| Cyma-Wejchenig et al. 2024 | Y | U | N | Y | N | N | Y | Y | 50.0 |
| DiFrancisco-Donoghue et al. 2025 | Y | Y | U | N | Y | Y | U | Y | 75 |
| DiFrancisco-Donoghue et al. 2022 | Y | Y | Y | Y | Y | Y | Y | N | 87.5 |
| Giakoni-Ramírez et al. 2021 | Y | U | Y | Y | N | N | Y | Y | 62.5 |
| Giakoni-Ramírez et al. 2022 | Y | Y | N | Y | Y | N | Y | Y | 75 |
| Goh et al. 2019 | Y | U | N | N | N | N | Y | Y | 37.5 |
| Han et al. 2012 | Y | U | Y | Y | Y | Y | Y | Y | 87.5 |
| Kang et al. 2020 | Y | Y | Y | Y | Y | Y | Y | Y | 100 |
| Ketelhut et al. 2023 | Y | U | Y | Y | Y | Y | Y | Y | 87.5 |
| Kidcaff et al. 2025 | Y | Y | U | Y | Y | Y | U | Y | 75 |
| Kulecka 2023 | Y | U | Y | Y | N | N | Y | Y | 62.5 |
| Lam et al. 2022 | U | U | Y | Y | N | N | N | Y | 37.5 |
| Lee et al. 2020 | U | U | Y | Y | Y | Y | Y | Y | 75 |
| Luts 2026 | U | Y | U | Y | N | N | Y | N | 37.5 |
| Madden and Harteveld 2021 | U | U | N | Y | N | N | Y | Y | 37.5 |
| Mancı and Özdalyan 2023 | Y | U | N | Y | N | N | Y | Y | 50 |
| Mancı et al. 2024 | Y | U | N | Y | Y | Y | Y | N | 62.5 |
| Ohno 2022 | U | U | N | Y | Y | Y | Y | Y | 62.5 |
| Onishchenko et al. 2025 | Y | Y | N | Y | Y | Y | U | Y | 75 |
| Overå et al. 2024 | Y | Y | N | Y | N | N | N | Y | 50 |
| Ren and Nie 2023 | Y | Y | N | Y | Y | N | Y | Y | 75 |
| Rudolf et al. 2020 | U | Y | N | N | N | N | Y | Y | 37.5 |
| Sand Hansen et al. 2025 | Y | Y | N | Y | N | N | Y | Y | 62.5 |
| Şarahman Kahraman et al. 2026 | Y | Y | U | Y | U | Y | Y | Y | 75 |
| Shan et al. 2023 | U | U | N | Y | Y | Y | Y | Y | 62.5 |
| Soares et al. 2022 | Y | Y | N | Y | N | N | Y | Y | 62.5 |
| Soffner et al. 2023 | Y | U | N | Y | N | N | Y | Y | 50 |
| Solmaz et al. 2025 | Y | Y | Y | Y | Y | Y | Y | Y | 100 |
| Tang et al. 2023 | Y | Y | N | Y | Y | Y | N | Y | 75 |
| Trotter et al. 2022 | N | U | Y | N | Y | Y | Y | Y | 62.5 |
| Total (Y%) | 69.4 | 52.8 | 36.1 | 86.1 | 50.0 | 47.2 | 83.3 | 88.9 | 64.2 |

**Items**

1. Were the criteria for inclusion in the sample clearly defined?
2. Were the study subjects and the setting described in detail?
3. Was the exposure measured in a valid and reliable way?
4. Were objective, standard criteria used for measurement of the condition?
5. Were confounding factors identified?
6. Were strategies to deal with confounding factors stated?
7. Were the outcomes measured in a valid and reliable way?
8. Was appropriate statistical analysis used?

N: negative answer (no), Y: positive answer (yes), U: unclear/partial (unclear)

**Supplementary Table 2 Leave-one-out sensitivity analysis of body mass index comparison**

| **Omitted Study** | ***k*** | **MD (95 % CI)** | ***p*** | ***I^2^*** |
| --- | --- | --- | --- | --- |
| Kulecka et al. 2023 | 12 | 0.46 (-0.07, 0.99) | 0.09 | 0% |
| Sand Hansen et al. 2025 | 12 | 0.25 (-0.26, 0.75) | 0.34 | 0% |
| Lee et al. 2020 | 12 | 0.27 (-0.21, 0.75) | 0.27 | 0% |
| Ketelhut et al. 2023 | 12 | 0.16 (-0.37, 0.69) | 0.55 | 0% |
| Cueva-Reguer et al. 2023 | 12 | 0.22 (-0.26, 0.70) | 0.37 | 0% |
| Mancı et al. 2024 | 12 | 0.33 (-0.14, 0.81) | 0.17 | 0% |
| DiFrancisco-Donoghue et al. 2022 | 12 | 0.36 (-0.12, 0.85) | 0.14 | 0% |
| Luts 2026 | 12 | 0.34 (-0.14, 0.82) | 0.16 | 0% |
| DiFrancisco-Donoghue et al. 2025 | 11 | 0.31 (-0.17, 0.80) | 0.21 | 1% |
| Cyma-Wejchenig et al. 2024 | 12 | 0.29 (-0.19, 0.77) | 0.24 | 0% |
| Ren and Nie 2023 | 12 | 0.28 (-0.21, 0.77) | 0.27 | 0% |
| Onishchenko et al. 2025 | 12 | 0.21 (-0.28, 0.71) | 0.40 | 0% |

**Supplementary Table 3 Leave-one-out sensitivity analysis of physical activity comparison**

| **Omitted Study** | ***k*** | **SMD (95 % CI)** | ***p*** | ***I^2^*** |
| --- | --- | --- | --- | --- |
| Kulecka et al. 2023 | 5 | -0.25 (-0.67, 0.18) | 0.25 | 70% |
| Ketelhut et al. 2023 | 5 | -0.27 (-0.69, 0.14) | 0.19 | 69% |
| DiFrancisco-Donoghue et al. 2022 | 5 | -0.04 (-0.23, 0.15) | 0.71 | 7% |
| Cyma-Wejchenig et al. 2024 | 5 | -0.37 (-0.47, 0.23) | 0.49 | 62% |
| Trotter et al. 2022 | 5 | -0.29 (-0.71, 0.12) | 0.17 | 64% |
| Mancı et al. 2024 | 5 | -0.53 (-0.59, 0.12) | 0.20 | 70% |

**Supplementary Table 4 Leave-one-out sensitivity analysis of anxiety comparison**

| **Omitted Study** | ***k*** | **SMD (95 % CI)** | ***p*** | ***I^2^*** |
| --- | --- | --- | --- | --- |
| Kang et al. 2020 | 3 | -0.02 (-0.17, 0.14) | 0.85 | 0% |
| Chaiwiang and Koo-Akarakul 2024 | 3 | -0.15 (-0.59, 0.29) | 0.50 | 78% |
| Lee et al. 2020 | 3 | -0.20 (-0.52, 0.12) | 0.27 | 73% |
| Soareset al. 2020 | 3 | -0.14 (-0.63, 0.34) | 0.55 | 77% |

**Supplementary Table 5 Leave-one-out sensitivity analysis of depression comparison**

| **Omitted Study** | ***k*** | **SMD (95 % CI)** | ***p*** | ***I^2^*** |
| --- | --- | --- | --- | --- |
| Chaiwiang and Koo-Akarakul 2024 | 4 | -0.01 (-0.07, 0.99) | 0.94 | 81% |
| Han et al. 2012 | 4 | -0.04 (-0.29, 0.21) | 0.75 | 81% |
| Lee et al. 2020 | 4 | -0.13 (-0.17, -0.10) | < 0.001 | 0% |
| Soareset al. 2020 | 4 | 0.09 (-0.26, 0.44) | 0.62 | 77% |
| Overa et al. 2024 | 4 | 0.22 (-0.26, 0.70) | 0.37 | 0% |

**Supplementary Table 6 Leave-one-out sensitivity analysis of stress comparison**

| **Omitted Study** | ***k*** | **SMD (95 % CI)** | ***p*** | ***I^2^*** |
| --- | --- | --- | --- | --- |
| Lee et al. 2020 | 2 | 0.17 (-0.05, 0.39) | 0.14 | 35% |
| Chaiwiang and Koo-Akarakul 2024 | 2 | 0.08 (-0.10, 0.27) | 0.38 | 0% |
| Soareset al. 2020 | 2 | 0.26 (-0.02, 0.53) | 0.07 | 0% |

**Supplementary Table 7 Leave-one-out sensitivity analysis of sleep duration comparison**

| **Omitted Study** | ***k*** | **SMD (95 % CI)** | ***p*** | ***I^2^*** |
| --- | --- | --- | --- | --- |
| Lee et al. 2020 | 2 | -0.07 (-0.58, 0.44) | 0.79 | 50% |
| DiFrancisco-Donoghue et al. 2022 | 2 | 0.09 (-0.01, 0.19) | 0.07 | 0% |
| Tang et al. 2023 | 2 | -0.02 (-0.86, 0.82) | 0.07 | 66% |

**Supplementary Table 8 Leave-one-out sensitivity analysis of fat percentage comparison**

| **Omitted Study** | ***k*** | **MD (95 % CI)** | ***p*** | ***I^2^*** |
| --- | --- | --- | --- | --- |
| Ketelhut et al. 2023 | 6 | 4.89 (2.95, 6.83) | < 0.001 | 32% |
| Kulecka et al. 2023 | 6 | 3.37 (-0.08, 6.82) | 0.06 | 77% |
| Luts 2026 | 6 | 4.32 (1.02, 7.62) | 0.01 | 81% |
| DiFrancisco-Donoghue et al. 2025 | 5 | 3.15 (-0.14, 6.44) | 0.06 | 84% |
| DiFrancisco-Donoghue et al. 2022 | 6 | 3.58 (0.30, 6.85) | 0.03 | 82% |
| Onishchenko et al. 2025 | 6 | 3.27 (-0.08, 6.61) | 0.06 | 79% |

**Supplementary Table 9 Leave-one-out sensitivity analysis of correlation between participation and psychological well-being**

| **Omitted Study** | ***k*** | **Fisher’s Z (95 % CI)** | ***p*** | ***I^2^*** |
| --- | --- | --- | --- | --- |
| Goh et al. 2019 | 3 | -0.02 (-0.07, 0.04) | 0.57 | 0% |
| Shan et al. 2023 | 3 | -0.14 (-0.45, 0.17) | 0.39 | 92% |
| Soffner et al. 2023 | 3 | -0.14 (-0.45, 0.17) | 0.37 | 92% |
| Madden and Harteveld 2021 | 3 | -0.16 (-0.38, 0.06) | 0.16 | 92% |

**Supplementary Table 10 Leave-one-out sensitivity analysis of correlation between participation and gaming disorder**

| **Omitted Study** | ***k*** | **Fisher’s Z (95 % CI)** | ***p*** | ***I^2^*** |
| --- | --- | --- | --- | --- |
| Ohno 2022 | 4 | 0.28 (0.17, 0.40) | < 0.001 | 76% |
| Bäcklund et al. 2024 | 6 | 0.21 (0.10, 0.32) | < 0.001 | 79% |
| Cheng et al. 2023 | 6 | 0.20 (0.07, 0.75) | < 0.001 | 79% |
| Aggarwal et al. 2020 | 6 | 0.20 (0.10, 0.30) | < 0.001 | 79% |
| Solmaz et al. 2025 | 6 | 0.18 (0.11, 0.24) | < 0.001 | 30% |

**Supplementary Figure 1 Funnel plot for body mass index comparison
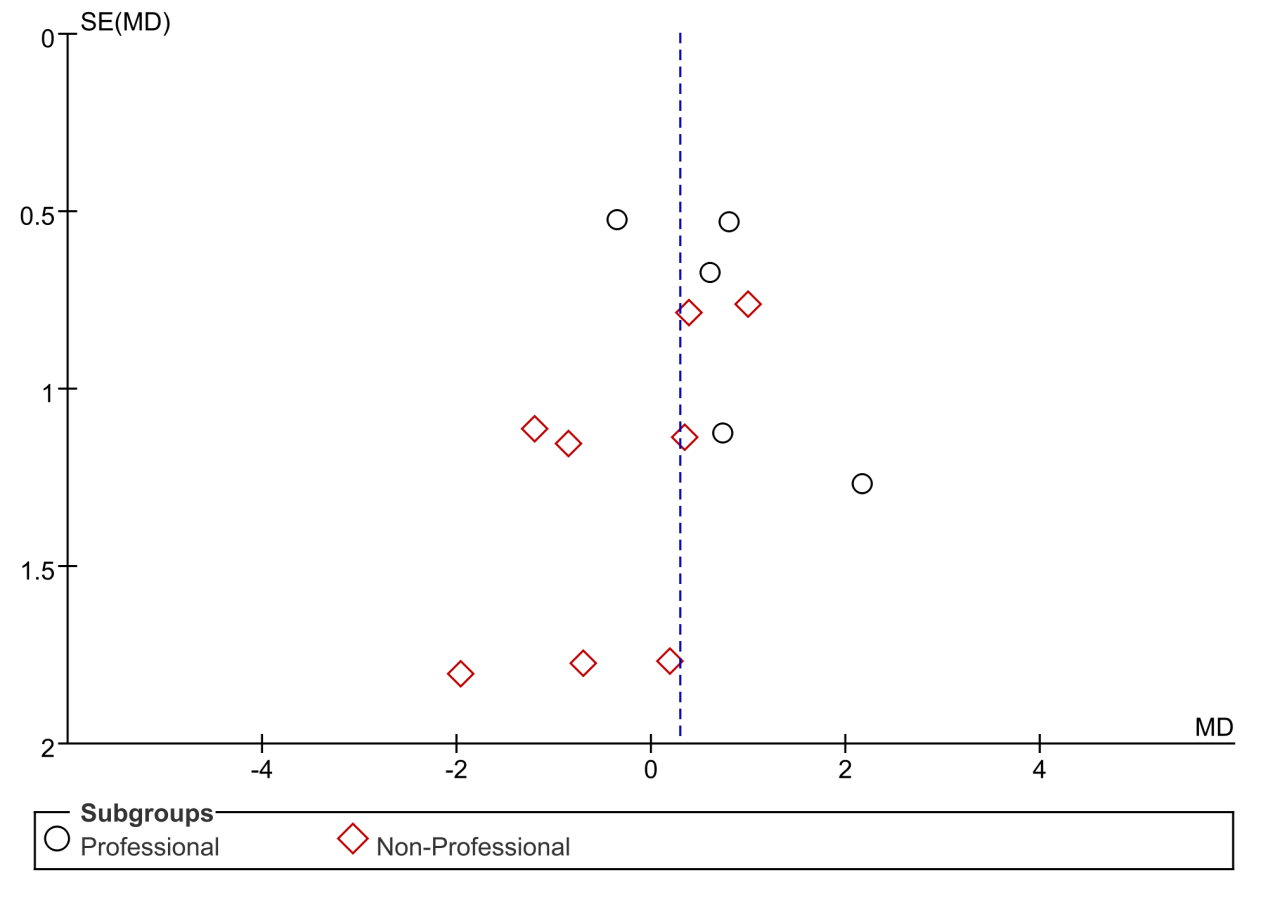
**

**Supplementary Figure 2 Funnel plot for physical activity comparison
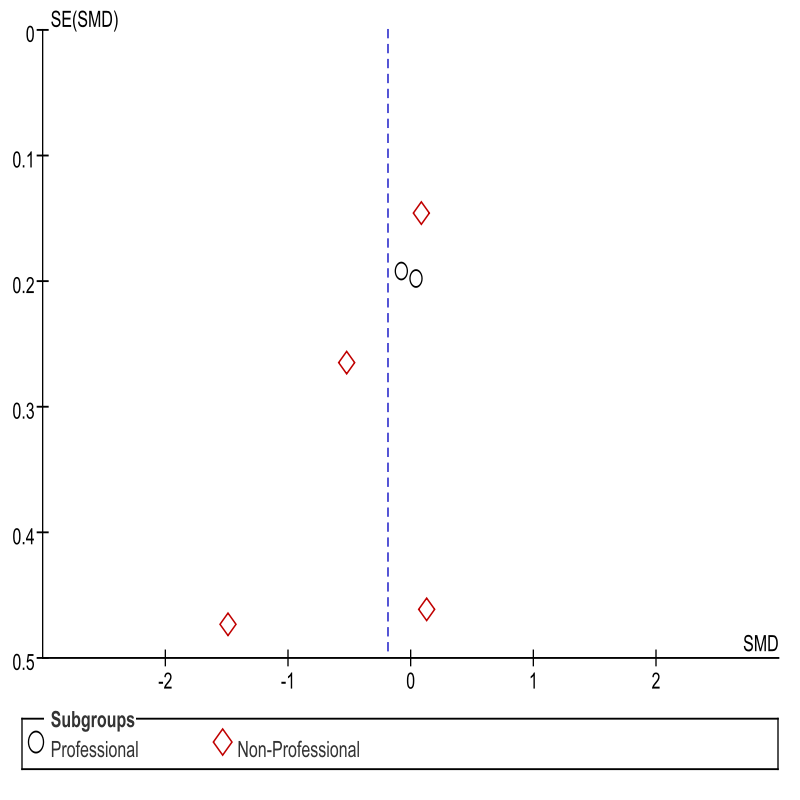
**

**Supplementary Figure 3 Funnel plot for depression comparison
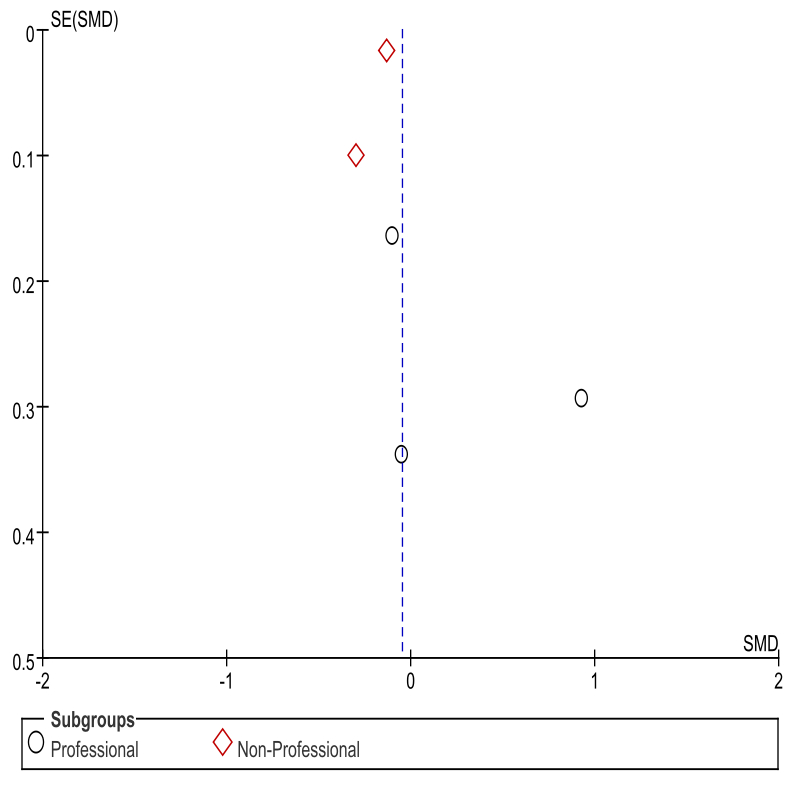
**

**Supplementary Figure 4 Funnel plot for anxiety comparison
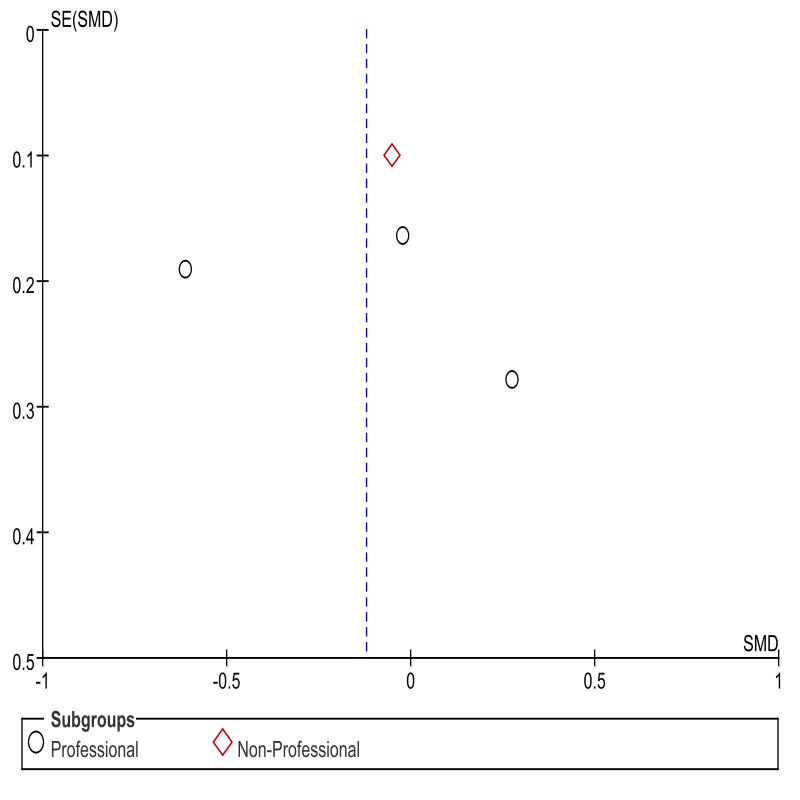
**

**Supplementary Figure 5 Funnel plot for stress comparison
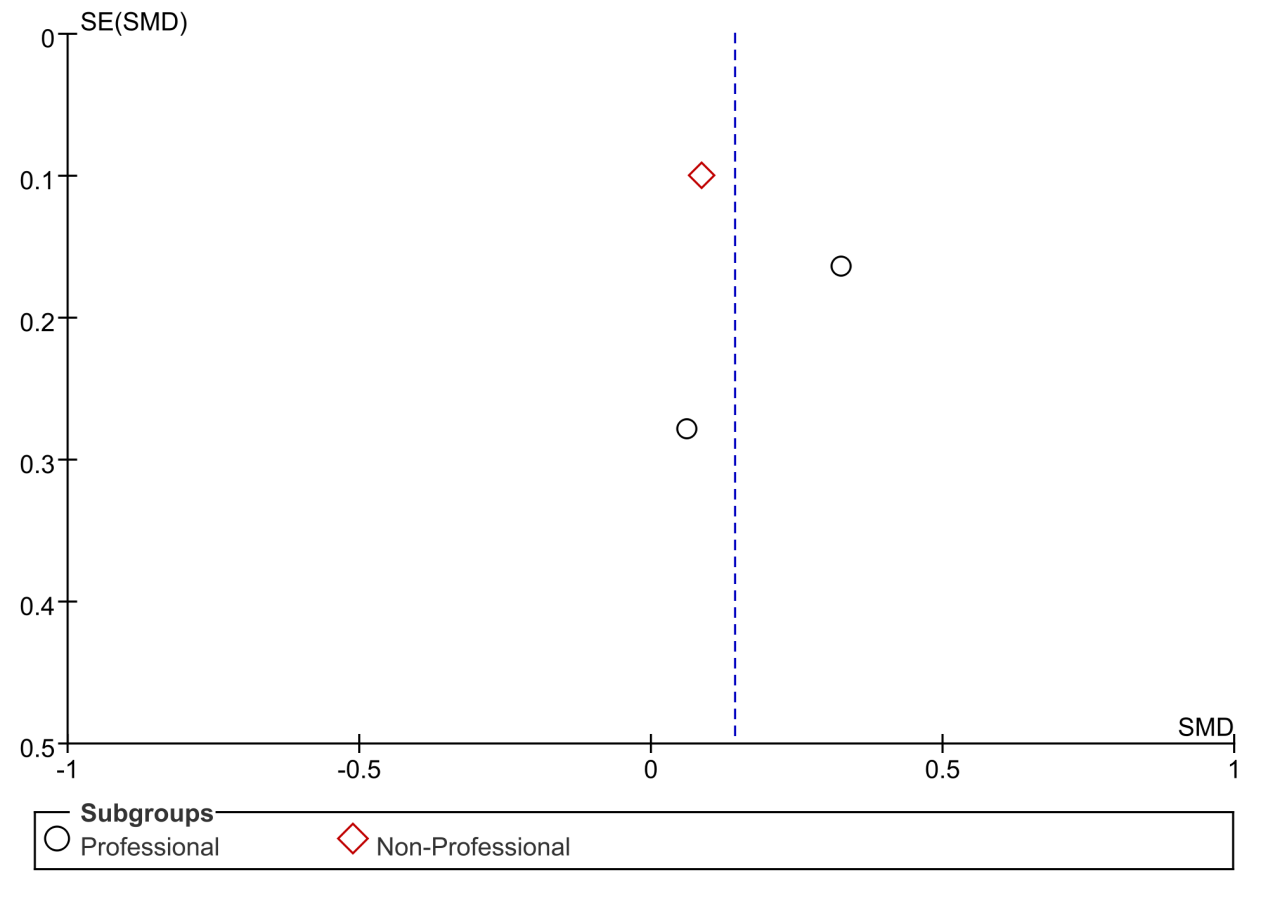
**

**Supplementary Figure 6 Funnel plot for sleep duration comparison
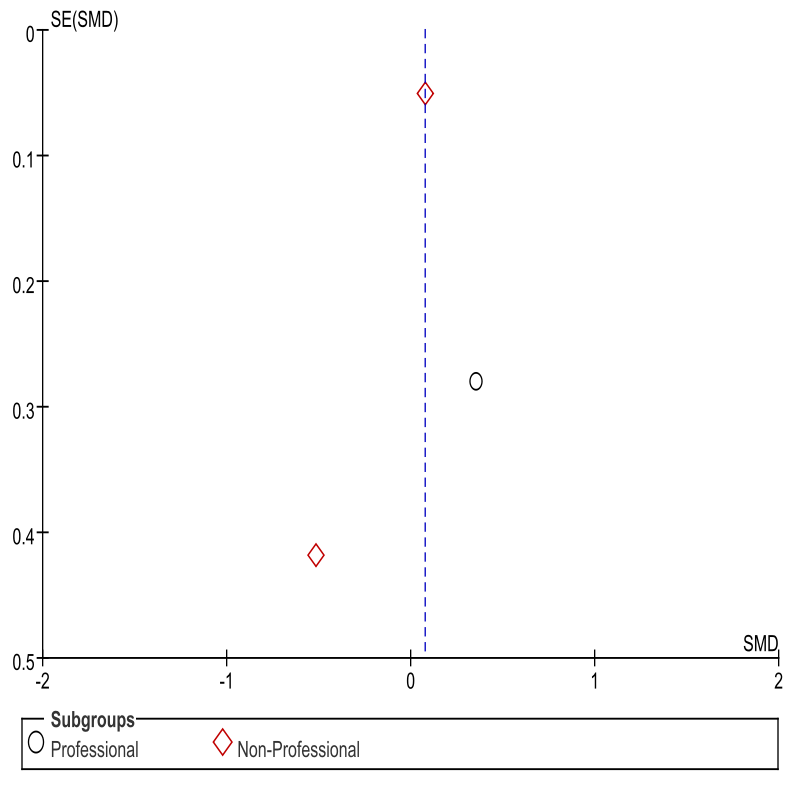
**

**Supplementary Figure 7 Funnel plot for fat percentage comparison
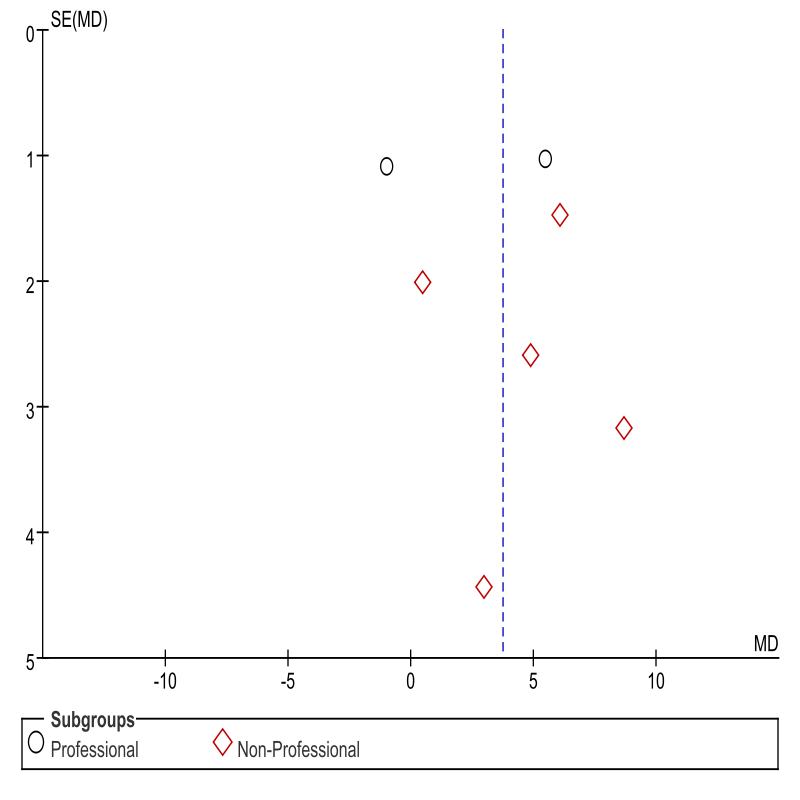
**

**Supplementary Figure 8 Funnel plot for grip strength comparison
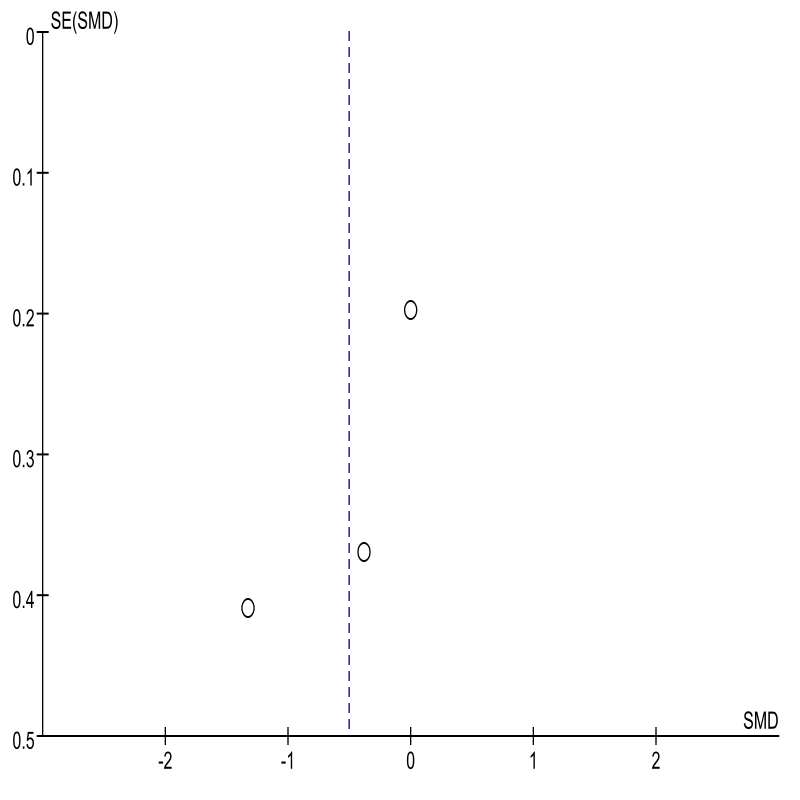
**

**Supplementary Figure 9 Funnel plot for the correlation between participation and psychological well-being
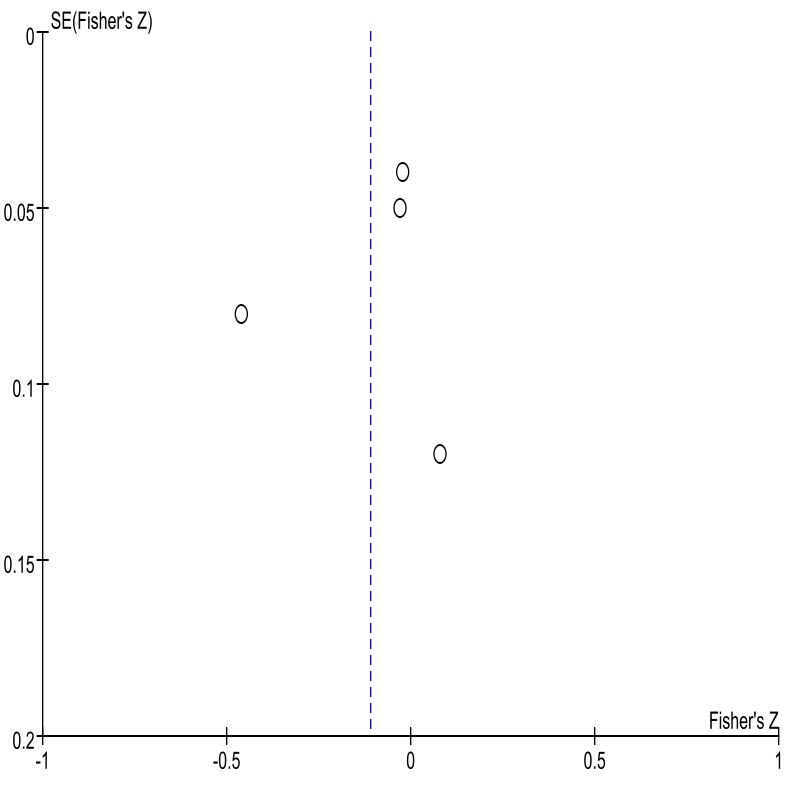
**

**Supplementary Figure 10 Funnel plot for the correlation between participation and gaming disorder
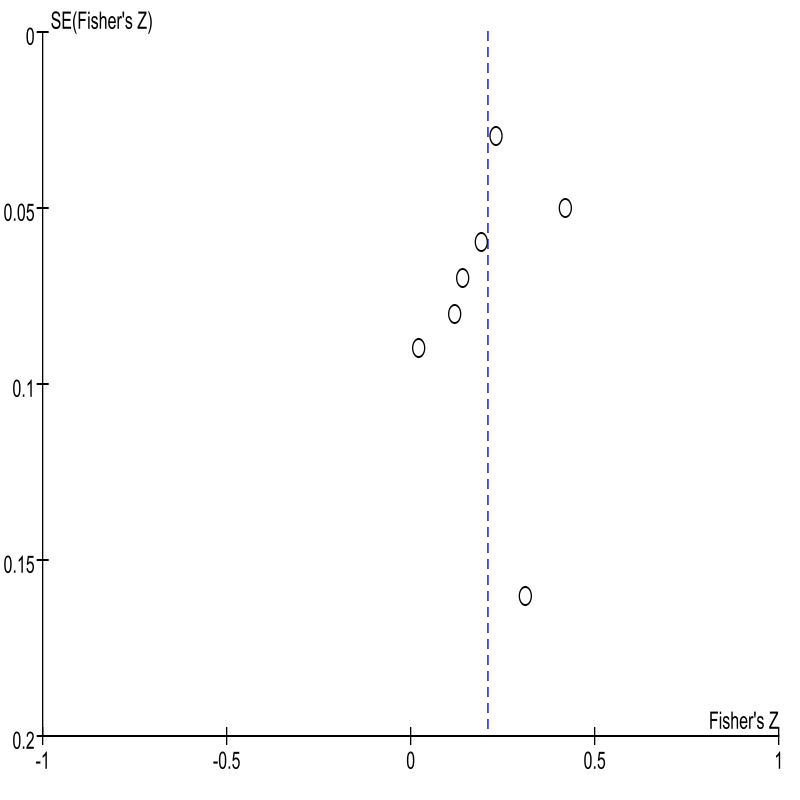
**

**Supplementary Figure 11 Funnel plot for the correlation between participation and body mass index
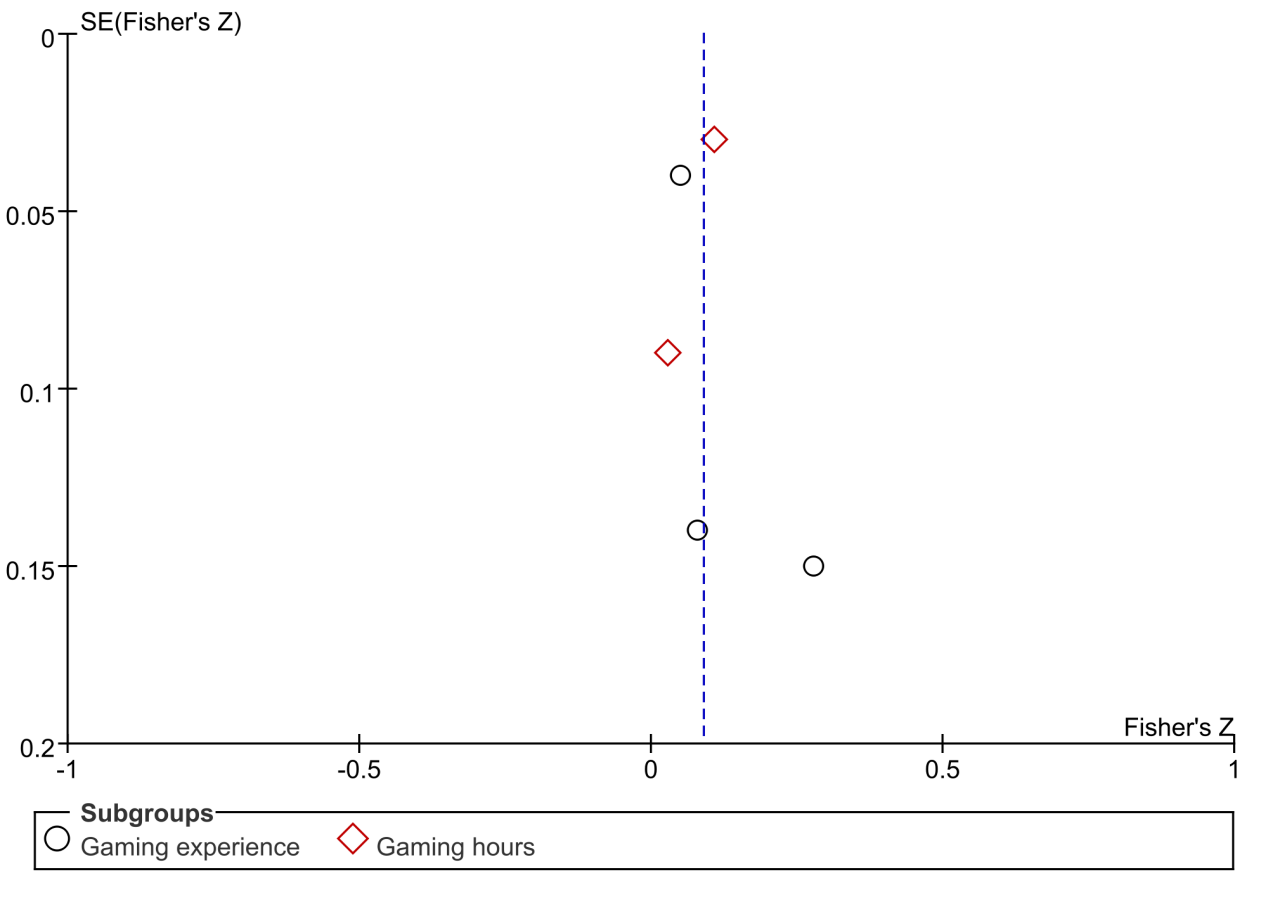
**

**Supplementary Figure 12 Funnel plot for the correlation between participation and sedentary behavior
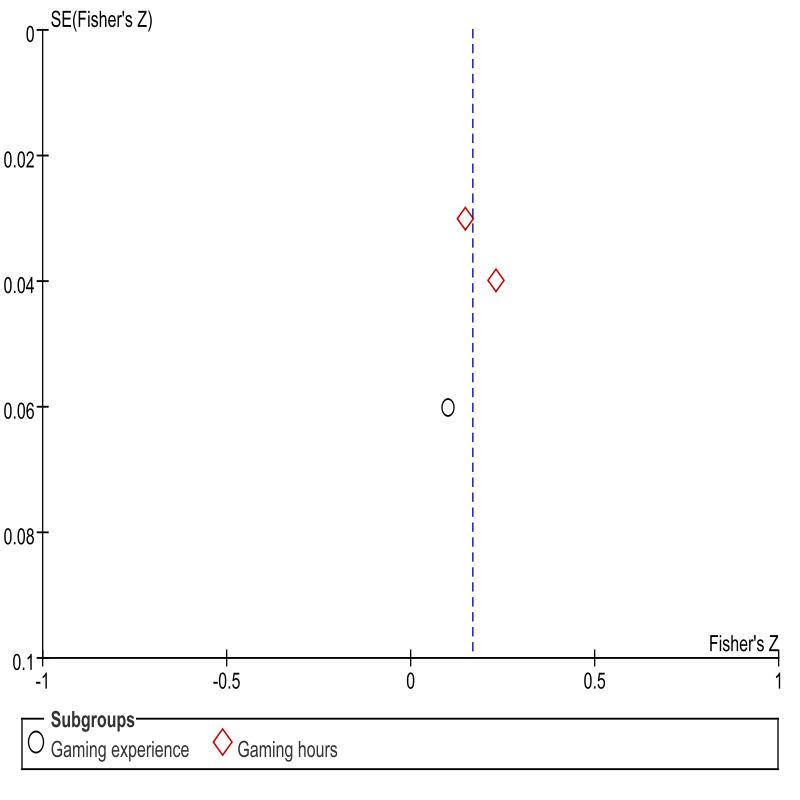
**

**Supplementary Figure 13 Funnel plot for the correlation between participation and physical activity
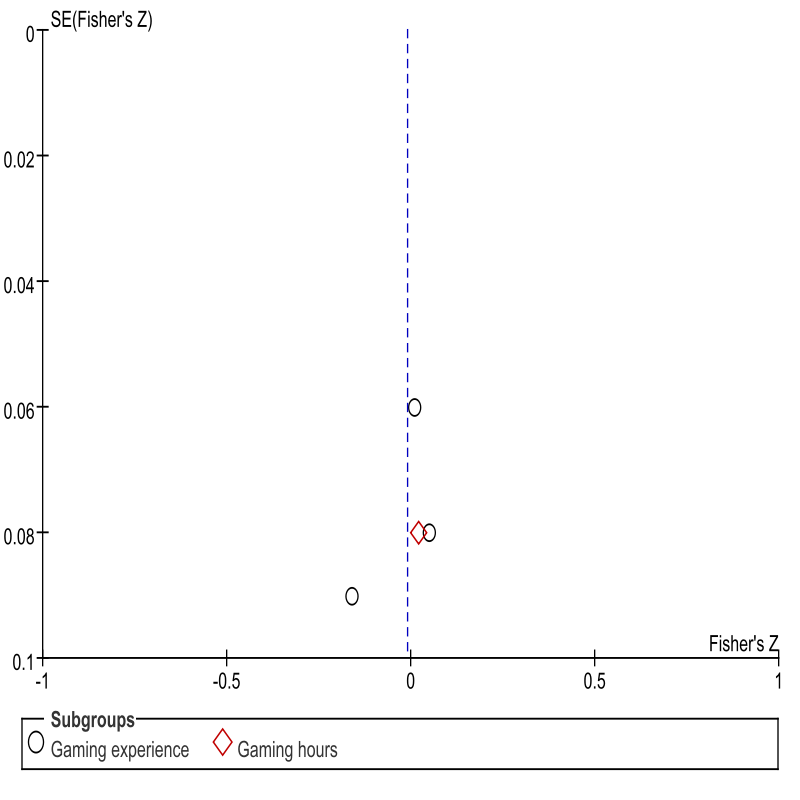
**

**Supplementary Figure 14 Funnel plot for the correlation between participation and stress
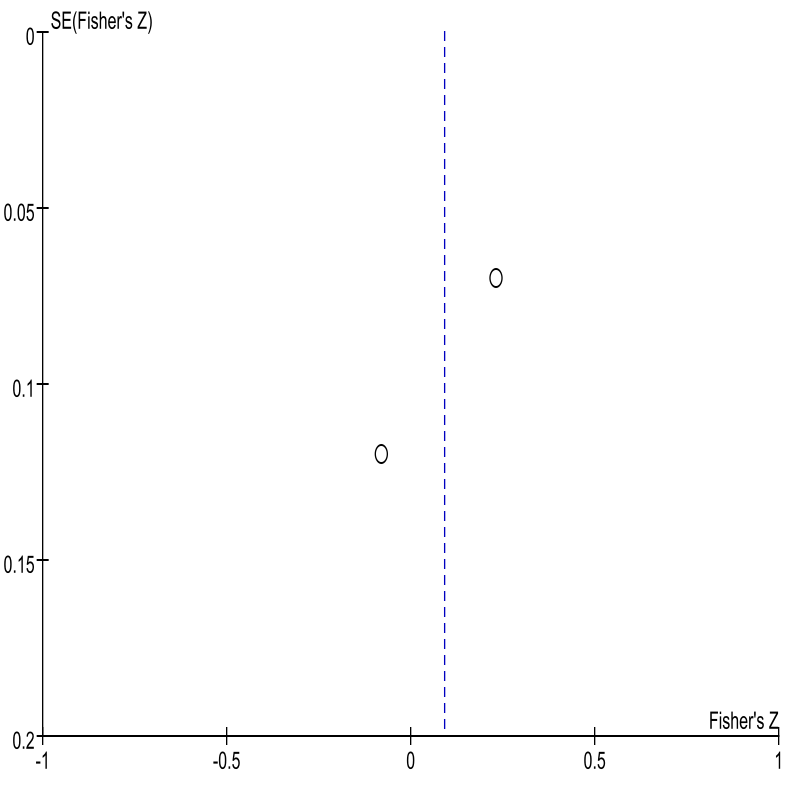
**

**Supplementary Figure 15 Funnel plot for the correlation between participation and sleep quality
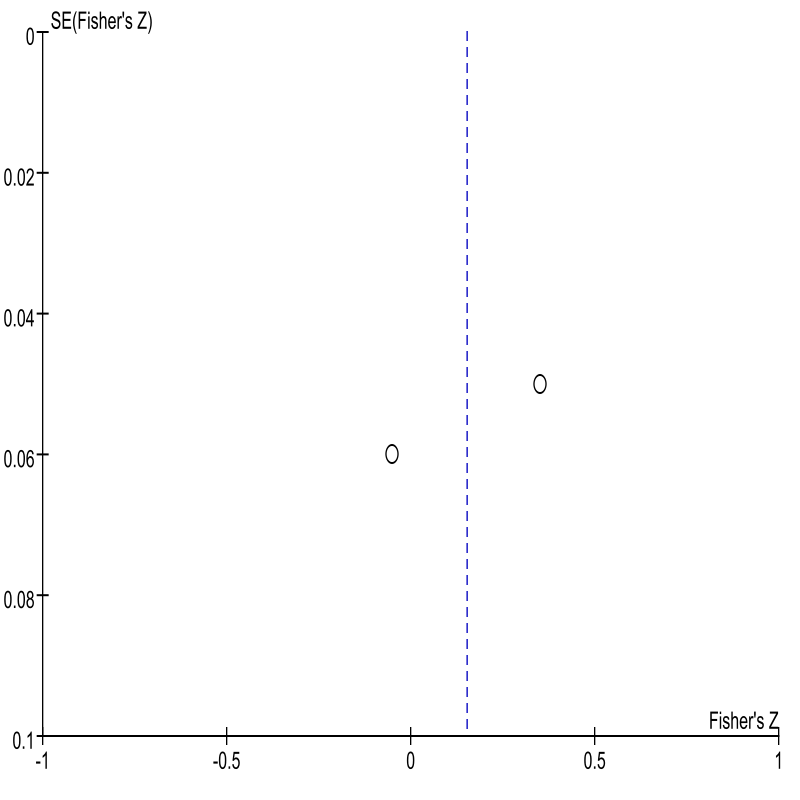
**
